# Tdrd6a and Tdrd6b are required together for germ plasm formation

**DOI:** 10.1242/dev.204545

**Published:** 2026-03-27

**Authors:** Alessandro Consorte, Joanna Michowicz, Fiona Carey, Martin M. Möckel, Yasmin El Sherif, Nadine Wittkopp, René F. Ketting

**Affiliations:** ^1^ Institute of Molecular Biology, Ackermannweg 4, 55128 Mainz, Germany; ^2^ International PhD Programme on Gene Regulation, Epigenetics & Genome Stability, 55128 Mainz, Germany; ^3^ University Mainz: Johannes Gutenberg University, Institute of Developmental Biology and Neurobiology, 55099 Mainz, Germany

**Keywords:** Zebrafish, Germ cell, Germ plasm, Tudor domain, Bucky ball

## Abstract

Germ cell specification is often driven by germ plasm (Gp), phase separation-based structures in the embryo, formed by maternal RNA and proteins. In the zebrafish, these oocyte-derived factors form a large structure known as the Balbiani body, which is also required for proper oocyte polarization. How the formation of these entities is regulated, especially in vertebrates, remains unclear. In this study, we show that two multi-Tudor proteins, Tdrd6a and Tdrd6b, are together required for Gp formation in zebrafish. Although the Balbiani body in the oocyte is affected in the absence of Tdrd6a, but not Tdrd6b, this does not affect oocyte functionality. In contrast, Gp is largely dispersed at the 4-cell stage, resulting in the absence of primordial germ cells during later development and sterility. Furthermore, we show that the Prion-like domain of Tdrd6b is relevant for Tdrd6b aggregation, as well as for its interaction with Buc, and that this is modulated by Tdrd6b Tudor domains. The implication of Tdrd6a and Tdrd6b in Gp stability is an important step in our understanding of how this phase-separated structure is controlled during development.

## Teaser

**Summary:** Maternal Tdrd6a and Tdrd6b proteins interact with Bucky ball and are essential for germ plasm formation, germ cell specification and fertility in zebrafish.

## INTRODUCTION

The germ line of a multicellular organism consists of a specific set of cells, the germ cells, which are essential for the fertility and the reproduction of the individual. Only these cells have the capability to form the zygote, a totipotent cell from which a whole organism can develop. Two major strategies have been identified for specifying the germ cells: induction and pre-formation (Extavour and Akam, 2003). The induction mode relies on signaling pathways, which act between cells of the developing embryo, to trigger a small group of cells to differentiate into primordial germ cells (PGCs). Pre-formation, instead, functions via the deposition of determinants (proteins and RNAs) expressed in the oocytes. Upon fertilization, these maternal determinants become enriched within a specific set of cells that will then take on PGC fate.

Although pre-formation mechanisms can differ between species, they all rely on phase separation-related mechanisms, resulting in the formation of germ plasm (Gp) (Boke et al., 2016; Bose et al., 2022; Brangwynne et al., 2009). In this study, we focus on Gp formation in the zebrafish (*Danio rerio*). In zebrafish oocytes, the protein Bucky ball (Buc) triggers the formation of a large amyloid-like structure, called the Balbiani body (Bb). This body lies at the vegetal side of the oocyte and becomes enriched with many determinants relevant for PGC specification (Boke et al., 2016; Bontems et al., 2009; Marlow and Mullins, 2008). With the growth and maturation of the oocyte, the Bb disperses into small patches at the vegetal cortex of the cell. After fertilization, Bb components migrate to the animal pole, where the embryo develops, and become organized into relatively small cytoplasmic droplets. With the start of the first two cell divisions, many of these small condensates align along the forming cleavage furrows of dividing cells, most notably at the distal end (close to the yolk). This process results in the formation of four clusters of droplets: these are referred to as Gp. By the onset of gastrulation, the Gp ends up in four groups of cells, driving their development as PGCs (Raz, 2003). Although we know much about the timed assembly and distribution of Gp, many proteins regulating these transitions remain unknown.

Buc is a known organizer of the Bb in zebrafish oocytes (Bontems et al., 2009; Dosch et al., 2004; Marlow and Mullins, 2008). Interestingly, Buc is a largely intrinsically disordered protein with a predicted Prion-like domain (PrLD) at its N terminus. This PrLD has been shown to be necessary to confer amyloid-like features to the Bb (Boke et al., 2016). This is also consistent with the observation of other phase-separation related studies in which intrinsically disordered proteins and PrLDs have been found to be relevant for the regulation of different condensates (Alberti et al., 2009; Banani et al., 2017; Kroschwald et al., 2015; Malinovska et al., 2013). The Bb, however, remains one of the few structures known thus far to show clear amyloid characteristics, while maintaining a dynamic nature: it disperses during later oogenesis and the Gp reassembles from many of its components during embryogenesis (Kar et al., 2025).

In previous work, we have shown how a multi-Tudor domain containing protein, Tdrd6a, interacts with Buc and affects its mobility (Roovers et al., 2018). The absence of Tdrd6a protein during zebrafish early development causes a reduction in the number of PGCs at the larval stages, accompanied by mild Gp defects. Analysis of the Buc–Tdrd6a interaction showed it was mediated through binding of methylated arginine/glycine (RG) motifs in Buc, presumably by the extended Tudor Domains (eTDs) of Tdrd6a. This study also revealed the presence of Tdrd6b, a paralog of Tdrd6a, in immunoprecipitations of both Buc and Tdrd6a. The latter interaction was shown to depend on the presence of Buc (Roovers et al., 2018).

In this work, we show that Tdrd6a and Tdrd6b both contribute to the stability of the embryonic Gp, and that they act redundantly to ensure PGC formation and fertility of the zebrafish. Furthermore, our data suggest that the Buc–Tdrd6b interaction is mediated the PrLD of Tdrd6b. Thus, our work provides deeper insights into the regulation of germ cell pre-formation mechanisms and describes a first mutant line of zebrafish (*tdrd6a;tdrd6b* double mutants) in which the embryonic Gp is fully destabilized during early development, without affecting embryonic viability and growth of the animal.

## RESULTS

### Tdrd6b has seven extended Tudor domains and a Prion-like domain

In zebrafish, two Tdrd6 paralogs are encoded within the genome: *tdrd6a* (ENSDARG00000070052) on chromosome 20 and *tdrd6b* on chromosome 17 (ENSDARG00000089954) (Fig. S1A). Interestingly, both *tdrd6* loci of zebrafish share a large exon of over 5000 bp, encoding the majority of the final protein product, while all the other coding exons are below 200 bp in size. Furthermore, both *tdrd6-like* genes code for proteins of similar size and structure, harboring seven predicted eTDs, and both are expressed in the zebrafish germline and were identified as an interactor of Gp components in mass-spectrometry analyses of different protein pull-downs (Liu et al., 2022; Roovers et al., 2018). Unlike *tdrd6a*, *tdrd6b* has not been studied in further detail.

**Fig. 1.**
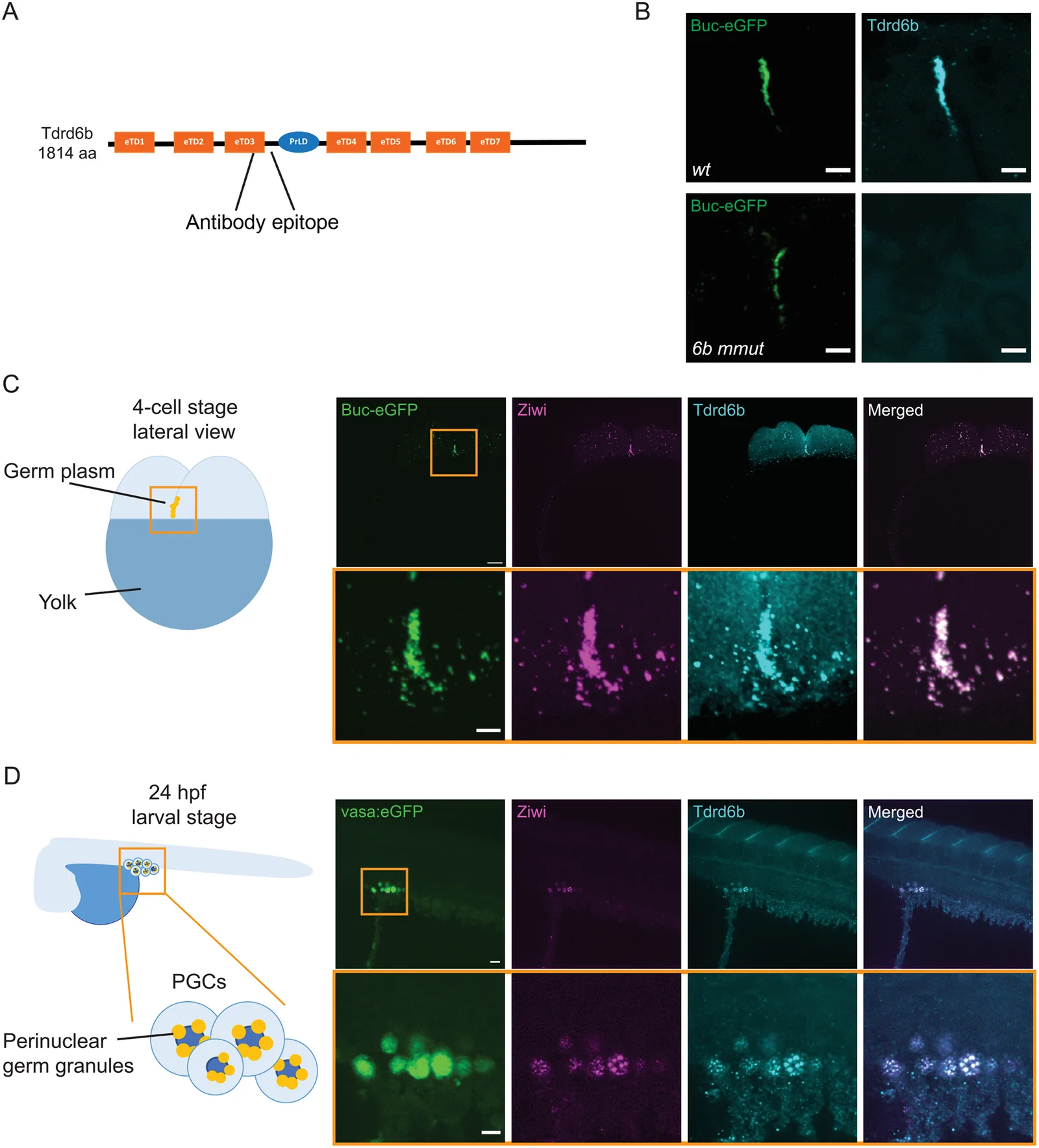
**Tdrd6b localization in zebrafish embryos.** (A) Scheme of Tdrd6b protein structure; orange boxes indicate Tudor domains (eTDs) and the blue oval the predicted prion-like domain (PrLD). (B) Validation of the specificity of the Tdrd6b antibody, showing its signal colocalizing with Buc-eGFP in *wt* embryos (top) but being absent in embryos collected from *tdrd6b^−/−^* mutant mothers. (C) Left: Schematic of the lateral view of a 4-cell-stage zebrafish embryo, with yellow Gp droplets distributed on the cleavage furrow. Right: Colocalization of Buc-eGFP (green), Ziwi (magenta) and Tdrd6b (cyan). Lower panels show the boxed area at higher magnification. (D) Left: Schematic of a 24 hpf zebrafish larva, with PGCs localized ventrally in proximity of the yolk (dark blue); yellow circles indicate perinuclear granules. Right: vasa:eGFP-positive PGCs, with colocalization of Tdrd6b (magenta) and Ziwi (cyan) in perinuclear granules Lower panels show the boxed area at higher magnification, showing PGCs. Scale bars: 20 µm (B); 50 µm (C, upper panels); 10 µm (D, upper panels); 5 µm (C,D, lower panels). Staining was carried out at least three times, with similar results.

*D. rerio tdrd6b* encodes a protein of 1814 amino acids in length that contains seven eTDs (Fig. 1A). When visualized by Alpha Fold predictions, the eTDs of Tdrd6b have the additional α-helices and β-sheets typical of eTDs (Fig. S1B) (Jumper et al., 2021; Liu et al., 2010a,b). This class of Tudor domains has often been found in the interaction with substrates containing methylated arginines. In eTDs, four residues form an aromatic cage within the β-barrel core structure, where one asparagine residue exhibits affinity for methylated substrates (Liu et al., 2010a,b). These are indeed found in the four C-terminal eTDs of Tdrd6b (Fig. S1C). However, the first three eTDs of Tdrd6b neither contain the aromatic cage, nor have the conserved asparagine, suggesting that these N-terminal eTDs of Tdrd6b could exhibit different substrate recognition (Fig. S1C). This organization is similar to that of Tdrd6a (Fig. S2), although the number of eTDs with and without an intact aromatic cage differs between the two proteins. Based on sequence homologies, it seems likely that specific eTDs duplicated or disappeared when Tdrd6a and Tdrd6b arose from a shared ancestral gene (Fig. S2A,C).

The presence of a predicted PrLD of 92 amino acids between the third and fourth eTD domains in Tdrd6b distinguishes it from Tdrd6a (Fig. S3). PrLDs are known to be involved in the regulation of phase-separation processes (Alberti et al., 2009; Shorter and Lindquist, 2005) and this region in Tdrd6b might be relevant in the regulation of Gp formation during zebrafish development.

### Tdrd6b is maternally inherited and localizes to Gp and perinuclear granules of PGCs

To investigate Tdrd6b expression and localization, we raised an antibody against a unique amino-acid sequence in rabbits and affinity-purified it. Immunofluorescence experiments on 4-cell-stage embryos derived from wild-type (*wt*) and *tdrd6b* mutant mothers (see below; note that the embryos obtained from the homozygous mutant mothers were themselves heterozygous) provided clear evidence of Tdrd6b localization to Gp-like structures (Fig. 1B). Indeed, we found that these structures were also positive for the Gp markers Buc, visualized by the transgenic line *Tg(buc:buc-eGFP)* (Riemer et al., 2015) and Ziwi (Piwil1) (Fig. 1C) (Houwing et al., 2007). Previously, we described that we found Tdrd6a protein surrounded Buc aggregates (Roovers et al., 2018). We did not see this for Tdrd6b, but we also did not see this for Tdrd6a in the current experiments (Fig. S4A). At 24 hours post-fertilization (hpf), we detected Tdrd6b enrichment in PGCs marked by expression of the *Tg(vasa:eGFP)* reporter (Fig. 1D) (Krøvel and Olsen, 2002). In these PGCs, the signal localized to perinuclear granules in PGCs, highlighted by Ziwi (Fig. 1D) (Houwing et al., 2007). In oocytes, this antibody unfortunately gave cross-reactivity, and a specific signal could not be discerned. We note, however, that Tdrd6b (then called Tdrd6c) has been detected in freshly ovulated oocytes (Roovers et al., 2018), indicating that Tdrd6c is maternally provided. Altogether, these results show that Tdrd6b is present in the embryonic Gp of zebrafish and can still be found in PGCs at 24 hpf, a pattern that is very similar to that of Tdrd6a (Roovers et al., 2018).

### A transgenic line expressing a Tdrd6b-mKate2 fusion protein marks nuage and Gp

**Fig. 2.**
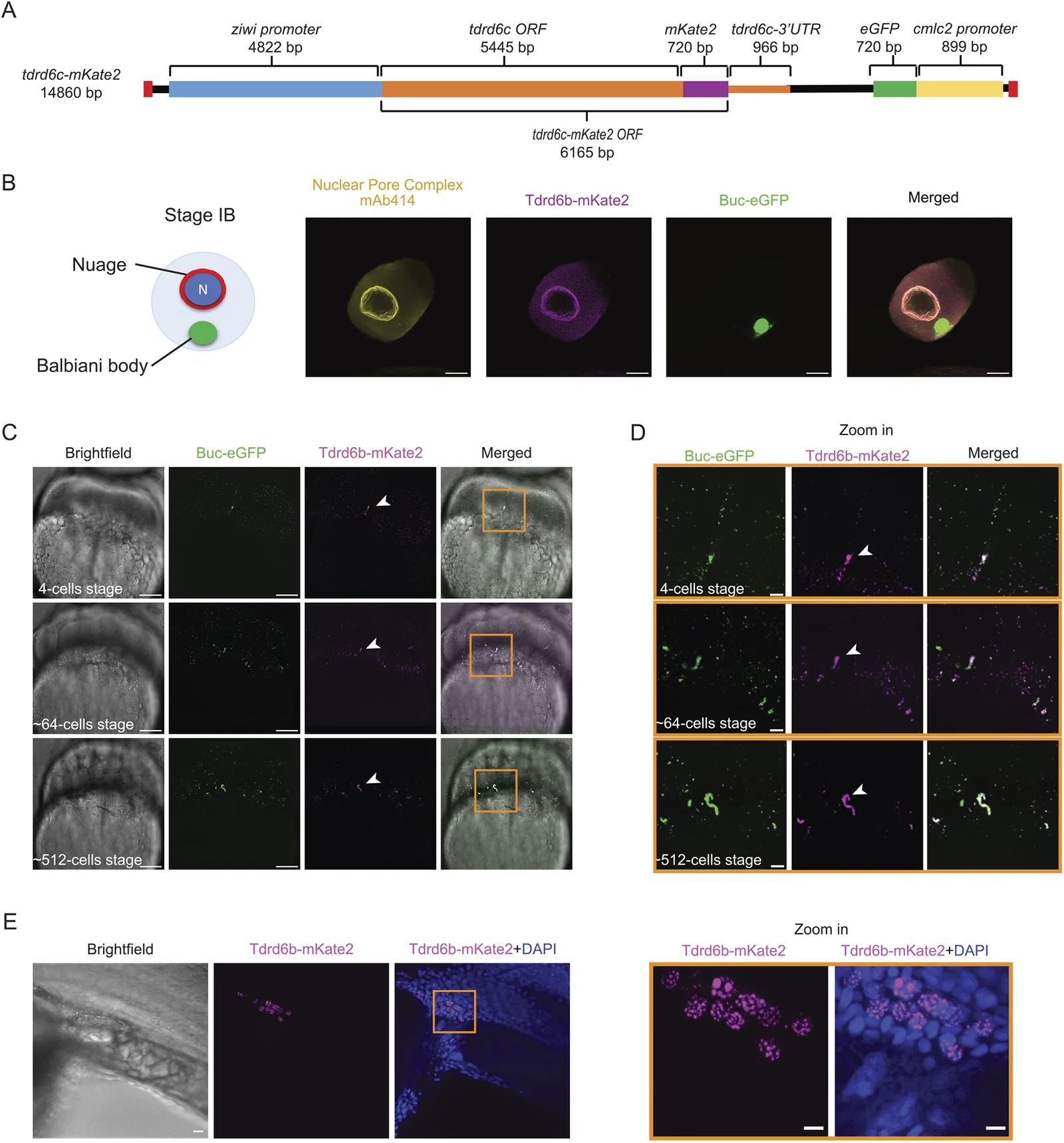
**Expression of the Tdrd6b-mKate2 transgene.** (A) Schematic of the *tdrd6b-mKate2* transgene inserted via Tol2-technology; in red are the Tol2 transposition sites; in black linker sequences. (B) Left: Schematic of a stage IB oocyte (nucleus in blue, perinuclear nuage in red and Bb in green). Right: Buc-eGFP (green)- and Tdrd6b-mKate2 (magenta)-expressing oocytes stained with mAb414 antibody, a nuclear pore marker (yellow). Similar experiments, with similar results, were carried out at least two more times. (C) Tdrd6b-mKate2 (magenta) localizes to the Gp (arrowheads) together with BucGFP (green), at different stages of embryogenesis. Similar results were obtained in at least two additional independent experiments. (D) Higher magnification images of the boxed areas in C. (E) Localization of Tdrd6b in perinuclear granules at 24 hpf (DAPI in blue) with higher magnification images of the boxed area shown on the right. Scale bars: 25 µm (B,D,E); 50 µm (C); 5 µm (E, right-hand panels). This perinuclear organization was seen in at least two more independent experiments.

To observe Tdrd6b localization and dynamics in living embryos, we generated a transgenic line expressing a Tdrd6b-mKate2 fusion protein using transposon-based Tol2-transgenesis technology (Kawakami, 2007). The designed transgene, *tg(ziwi:tdrd6b-mKate2-tdrd6b3′UTR)*, combines the *ziwi* promoter, the *tdrd6b* open reading frame (ORF), the mKate2 ORF and the *tdrd6b* 3′UTR (Fig. 2A). The *ziwi* promoter was chosen to drive expression during oogenesis (Leu and Draper, 2010) and the mKate2 fluorophore (Shcherbo et al., 2009) for combination with available GFP-expressing transgenic lines (Shcherbo et al., 2009).

Expression of Tdrd6b-mKate2 in oocytes revealed enrichment around the nucleus (nuage) and diffuse cytoplasmic staining (Fig. 2B). After fertilization, maternal Tdrd6b-mKate2 localized to Gp droplets marked by Buc-eGFP and enriched at the cleavage furrows of the dividing blastomeres (Fig. 2C,D, Movie 1). At later stages, Tdrd6b-mKate2 signal was detected in cells at the genital ridge of zebrafish larvae, which corresponds to the area where PGCs settle for further development (Fig. 2E). In particular, the Tdrd6b-mKate2 signal was enriched within granules around the PGC nuclei, termed perinuclear germ granules. These observations are in agreement with the results obtained with antibody staining and provide further evidence for the localization of Tdrd6b to embryonic Gp structures and to the nuage of zebrafish oocytes. We note, however, that this transgene could not rescue the *tdrd6a^−/−^;tdrd6b^−/−^* mutant sterility phenotype, even if Gp structures were sometimes partially rescued at the 4-cell stage (see below).

### Maternal Tdrd6b affects PGC formation

**Fig. 3.**
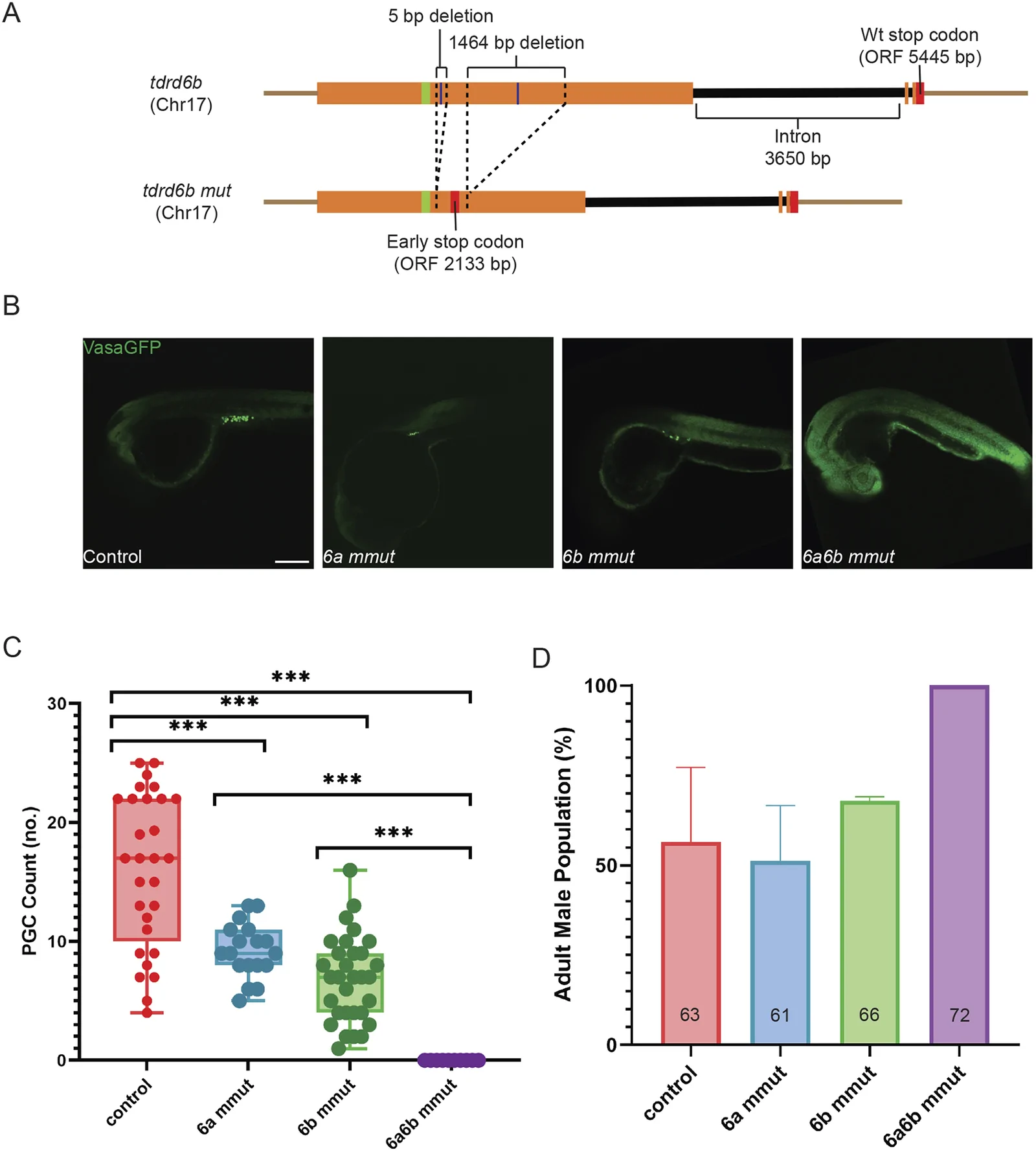
**PGC defects in offspring from *tdrd6a/b* mutants.** (A) Schematic of *tdrd6b* knockout allele. Black bars are intronic regions, orange boxes represent exons, red boxes stop codons, green box the epitope recognized by the Tdrd6b antibody and blue lines are targets of the two injected CRISPR RNAs. (B) vasa:eGFP-positive PGCs in embryos from mothers of different genotypes, showing reduction in *6a mmut* and *6b mmut* and total absence in *6a6b mmut*. Scale bar: 50 µm. Similar results were obtained in at least two additional independent experiments. (C) Quantification of PGC counts in embryos at 1 day post-fertilization. Numbers of animals scored: control, *n*=29; *6a mmut*, *n*=18; *6b mmut*, *n*=31; *6a6b mmut*, *n*=9. ***P<0.005 (two-sided Wilcoxon, no corrections for multiple comparisons). This experiment was repeated non-quantitatively at least three times. (D) Average percentage of males among offspring from females of the indicated genotypes; scored at 3 months of age. Numbers at the bottom of the columns refer to the total number of offspring from three different mothers. Control, *n*=63; *6a mmut*, *n*=61; *6b mmut*, *n*=66; *6a6b mmut*, *n*=72. Error bars represent s.d.

In order to create a *tdrd6b* mutant allele, we targeted its large exon with CRISPR-Cas9 using two guide RNAs (gRNAs) (Fig. 3A) (Hwang et al., 2013; Jinek et al., 2012). We successfully generated a *tdrd6b* knockout allele (*tdrd6b^mz69^*) that failed to express Tdrd6b, as its signal was not detectable after fertilization in embryos deriving from homozygous mutant mothers (Fig. 1B). Homozygous mutant *tdrd6b^−/−^* animals were viable under standard conditions, with fish exhibiting normal development and behavior. Also, these animals did not exhibit a bias towards male or female differentiation (Fig. 3D) and all tested adult fish were able to produce offspring.

To investigate whether PGC development was affected by the lack of maternally provided Tdrd6b protein, we combined the *tdrd6b^mz69^* allele with *Tg(vasa:eGFP)*, which is expressed at all stages of germ cell development. We found that embryos from *tdrd6b^−/−^* females, which we refer to as *tdrd6b* maternal mutants (*6b mmut*), had reduced numbers of PGCs at 24 hpf, compared to *wt* (Fig. 3B,C). This phenotype is similar to what was observed in *tdrd6a* maternal mutant embryos (*6a mmut*) (Roovers et al., 2018). In *tdrd6a* mutants, we did not detect upregulation of *tdrd6b* transcripts, or vice versa (Fig. S5A), suggesting that the weak phenotypes are not driven by transcriptional compensation of the other gene. We conclude that maternally provided Tdrd6a and Tdrd6b are individually involved in, but not essential for, PGC formation.

### Simultaneous absence of maternal Tdrd6a and Tdrd6b disrupts PGC formation

Next, we bred animals carrying mutations for both *tdrd6a* and *tdrd6b* and characterized their phenotypes. Like for the single mutants, *tdrd6a^−/−^;tdrrd6b^−/−^* (*6a6b*) double mutants obtained from heterozygous parents developed normally and were fertile. Strikingly, however, embryos obtained from *6a6b* double mutant females (*6a6b mmut* embryos) showed complete absence of PGCs at 24 hpf (Fig. 3B,C). This indicates that both maternal Tdrd6a and Tdrd6b contribute to PGC specification. The above-described transgene could not rescue this defect (Fig. S5B), indicating that some aspect of the expressed transgenic Tdrd6b protein is incompatible with full functionality.

Next, we raised *6a6b mmut* fish to adulthood and compared their growth and maturation to *wt* control populations and to *6a mmut* or *6b mmut* fish. These were collected on the same day and reared at the same density, aiming to minimize any stochastic influence from the environment in the different batches. After 3 months, all populations reached sexual maturity, with no superficial phenotype. However, when assessing sex ratios in each tank, we did not find any female fish in the *6a6b mmut* adult populations (Fig. 3D). Furthermore, 31 *6a6b mmut* adult males, obtained from different crosses, were tested for fertility and were unable to fertilize eggs upon mating with different *wt* females (Fig. S5C). The dissection of these males showed that *6a6b mmut* adult fish had severely under-developed gonads (Fig. S5D,E). This is consistent with previous studies that found that PGC loss leads to sterility and a strong bias towards male differentiation in zebrafish (D'Orazio et al., 2021; Gross-Thebing et al., 2017; Houwing et al., 2007; Siegfried and Nüsslein-Volhard, 2008; Weidinger et al., 2003). From these observations, we conclude that maternal Tdrd6a and Tdrd6b act redundantly in the process of PGC specification and that their simultaneous absence prevents the formation of germ cells during early development, resulting in sterility.

### Loss of Gp in absence of maternal Tdrd6a and Tdrd6b

**Fig. 4.**
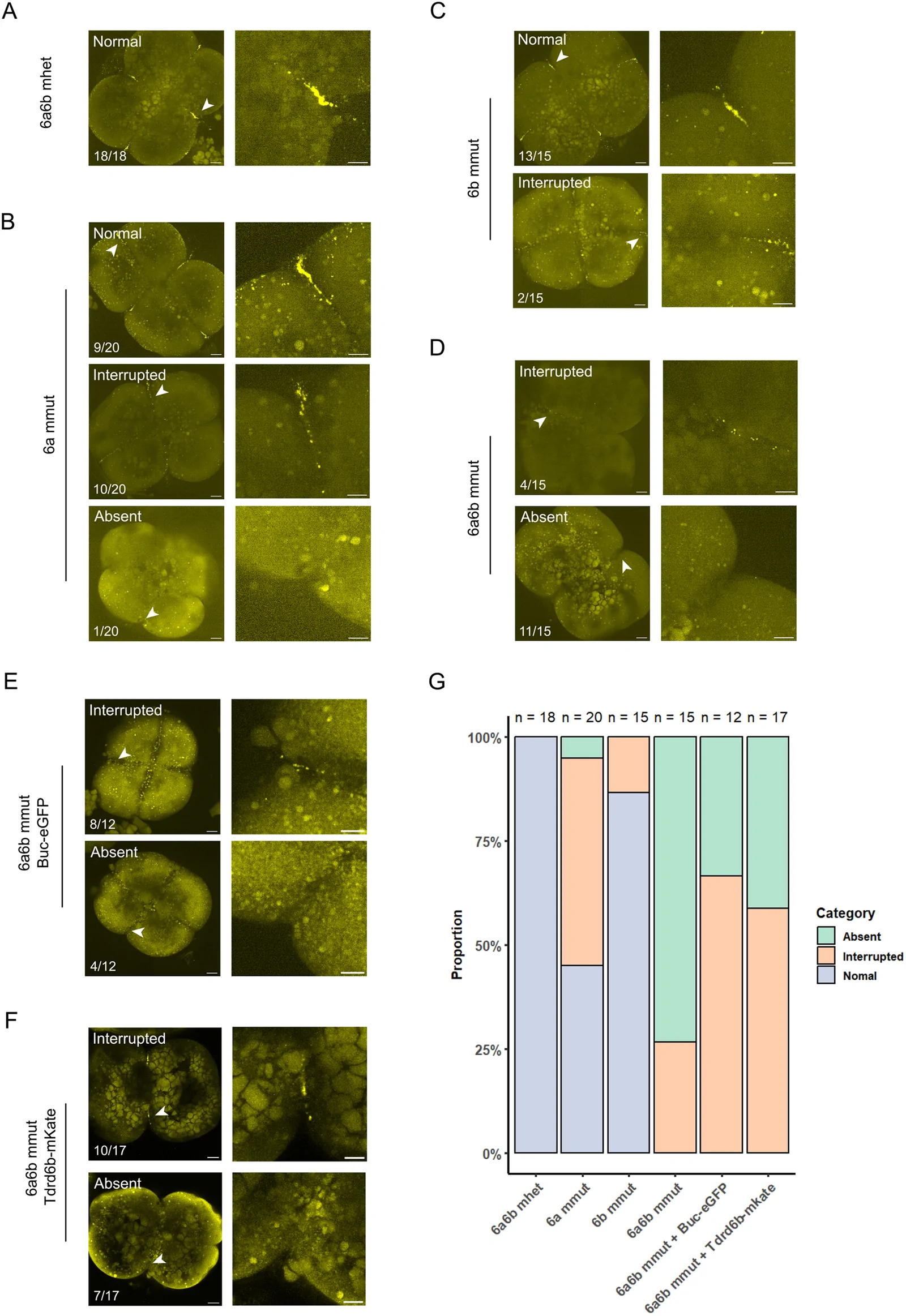
**Loss of Gp in the absence of maternal Tdrd6a and Tdrd6b.** (A-F) Gp, marked by *vasa* mRNA at the 4-cell embryo stage, from females of the indicated genotypes (maternal mutants). Embryos are categorized as ‘Normal’, ‘Interrupted’ or ‘Absent’ based on the continuity and integrity of the *vasa* mRNA localization pattern. For each genotype, the left panel shows a representative image of a 4-cell embryo, and the right panel shows a corresponding magnified region of the Gp marked with the arrowhead. Scale bar: 50 μm (left panels); 25 μm (right panels). (G) Bar graph summarizing the data as proportional distributions for each genotype. The analyzed number of embryos is given at the top of the bars. Embryos were obtained from clutch matings with at least three females. Similar results were obtained in two additional independent experiments. mhet, maternal heterozygous.

We addressed Gp formation in presence or absence of Tdrd6a or Tdrd6b by performing single-molecule fluorescence *in situ* hybridization (smFISH) against *vasa* (*ddx4*). We compared Gp structures of embryos from mothers with various genotypes, taking *tdrd6a^+/−^;tdrd6b^+/−^* double heterozygous mothers as wild-type baseline. These typically showed strong, continuous Gp signals at the cleavage planes of the 4-cell-stage embryo (Fig. 4A,G). Consistent with previous work on Tdrd6a (Roovers et al., 2018), more than half of the embryos from *tdrd6a^−/−^;tdrd6b^+/−^* mothers displayed defects (Fig. 4B,G). These defects ranged from interrupted Gp (detectable but discontinuous Gp structures) to undetectable Gp. Embryos from *tdrd6a^+/−^;tdrd6b^−/−^* mutant mothers tended to display weaker effects on Gp: they were less frequent and we did not observe absence of Gp (Fig. 4C,G). In contrast, embryos produced by homozygous *tdrd6a^−/−^;tdrd6b^−/−^* double mutant females had severely disrupted Gp (Fig. 4D,G). In more than 50% of the embryos, we could not detect Gp at all, while in the rest Gp was always interrupted. No *wt*-like Gp was detected. Maternal presence of the Tdrd6b-mKate transgene partially restored Gp, as evidenced by a decrease in embryos without any Gp (Fig. 4E,G). While this small effect was not enough to rescue germ cell formation defects (see above; Fig. S5B), it does suggest that the Tdrd6b-mKate is at least partially functional. Curiously, presence of the Buc-eGFP transgene showed a similar trend (Fig. 4F,G). In embryos from double mutants with *Buc-eGFP*, Buc-eGFP and endogenous Buc were only detected when *vasa* was also present and were absent in embryos lacking vasa clustering at the cleavage planes (Fig. S4B). Interestingly, in *6a6b mmut* double mutants expressing *Tdrd6b-mKate*, we observed different scenarios. When *vasa* was observed to cluster to some extent at the cleavage planes, we also detected Tdrd6b-mKate and Buc at these sites. When we did not detect *vasa*, Buc was absent while Tdrd6b-mKate was found spread all over the cleavage plane and not focused on the distal ends of the cleavage planes (Fig. S4B). Our interpretation of these observations is that Tdrd6b-mKate can localize to the cleavage planes independently of Buc or mRNPs, but that proper Gp formation at the distal sites also requires Buc and messenger ribonucleoproteins (mRNPs). These observations indicate that Tdrd6b-mKate can localize to the Gp but is not sufficient to recruit Buc or Gp RNAs such as *vasa*. We conclude that Tdrd6 activity (Tdrd6a and Tdrd6b combined) is essential for formation of Gp, and that Tdrd6a and Tdrd6b can at least partially compensate for the other's absence in forming Gp.

### The Balbiani body is not significantly affected by Tdrd6b

**Fig. 5.**
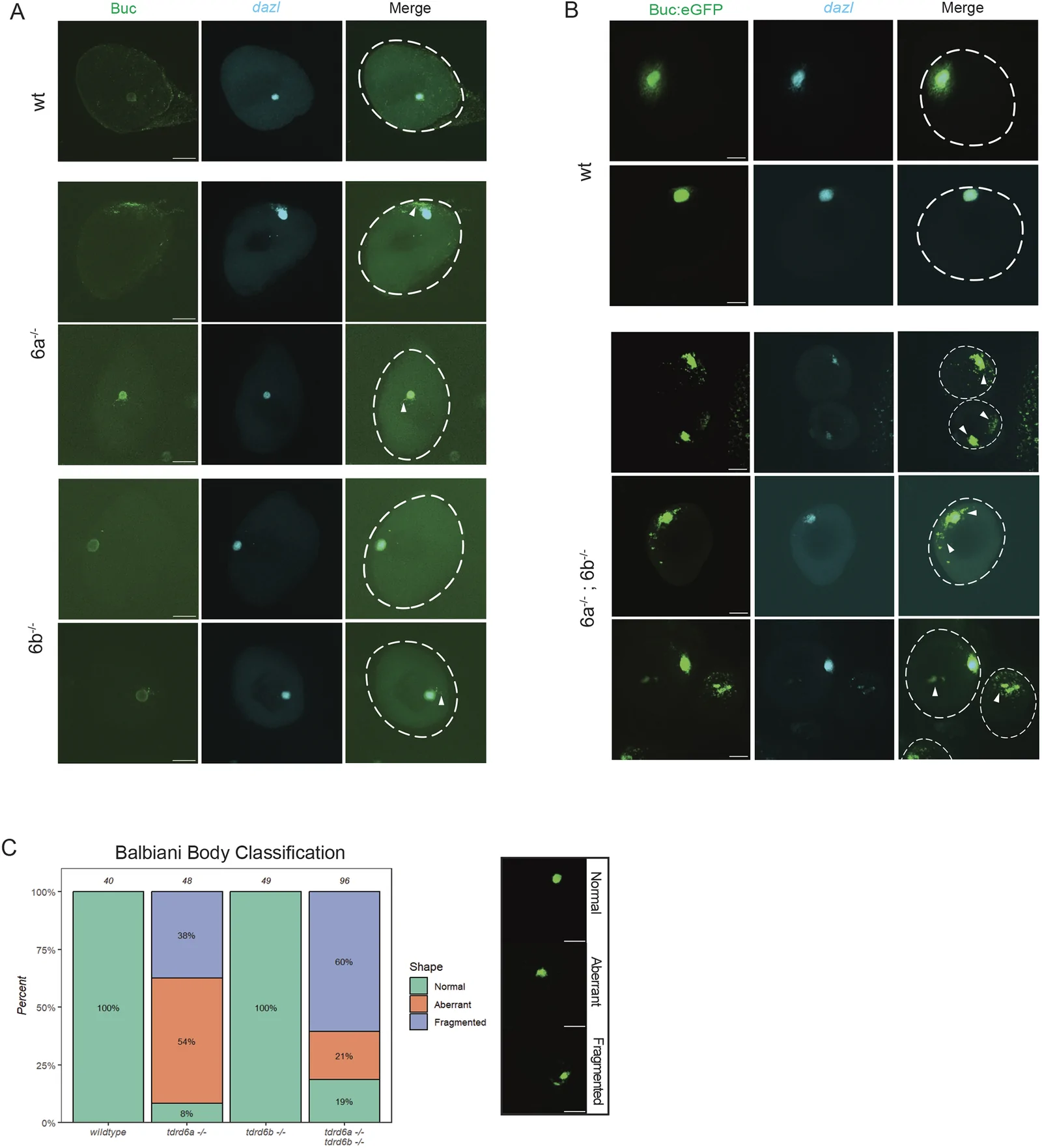
**Bb defects in *6a6b* double-mutant females.** (A) Representative images of Balbiani body (Bb) formation and *dazl* RNA localization in *wt*, *tdrd6a* mutant and *tdrd6b* mutant oocytes. Oocytes were analyzed by smFISH for *dazl* RNA together with immunostaining using a homemade anti-Buc antibody. Arrowheads indicate abnormalities. (B) *wt* and *tdrd6a^−/−^;tdrd6b^−/−^* double-mutant oocytes in a Buc:eGFP background. Arrowheads indicate abnormalities. Dashed lines in A,B encircle individual oocytes. (C) Quantification of Bb integrity in *wt* and mutant oocytes, obtained from two females. All data stem from strains carrying the Buc:eGFP transgene, and eGFP was used to visualize the Bb. Values above bars indicate total number of stage I oocytes analyzed. Similar results were obtained in two additional independent experiments. Examples of each category are shown on the right. Scale bars: 20 μm.

We previously described Bb defects in *tdrd6a* single mutant females. In our current analyses, using smFISH against *dazl* and Buc immunofluorescence we detected similar defects again in *tdrd6a* mutant oocytes. About 54% showed what we called an ‘aberrant’ phenotype: Buc-positive material appears to ‘leak’ from the Bb (Fig. 5A,C) and 38% were found to be fragmented: multiple larger Buc-positive fragments were found within the oocyte (Fig. 5A,C). *Tdrd6b* mutant oocytes did not display such defects (Fig. 5A,C). Double-mutant oocytes showed aberrant and fragmented Bb structures at frequencies similar to those in *tdrd6a* single mutants (Fig. 5B,C). The perinuclear nuage, as judged from Zili (Piwil2) staining, was not affected (Fig. S6). We conclude that Tdrd6a contributes to Bb formation and that a contribution of Tdrd6b to Bb formation is not detectable in our experiments.

### The Tdrd6b PrLD triggers protein interaction and self-assembly in BmN4 cells

**Fig. 6.**
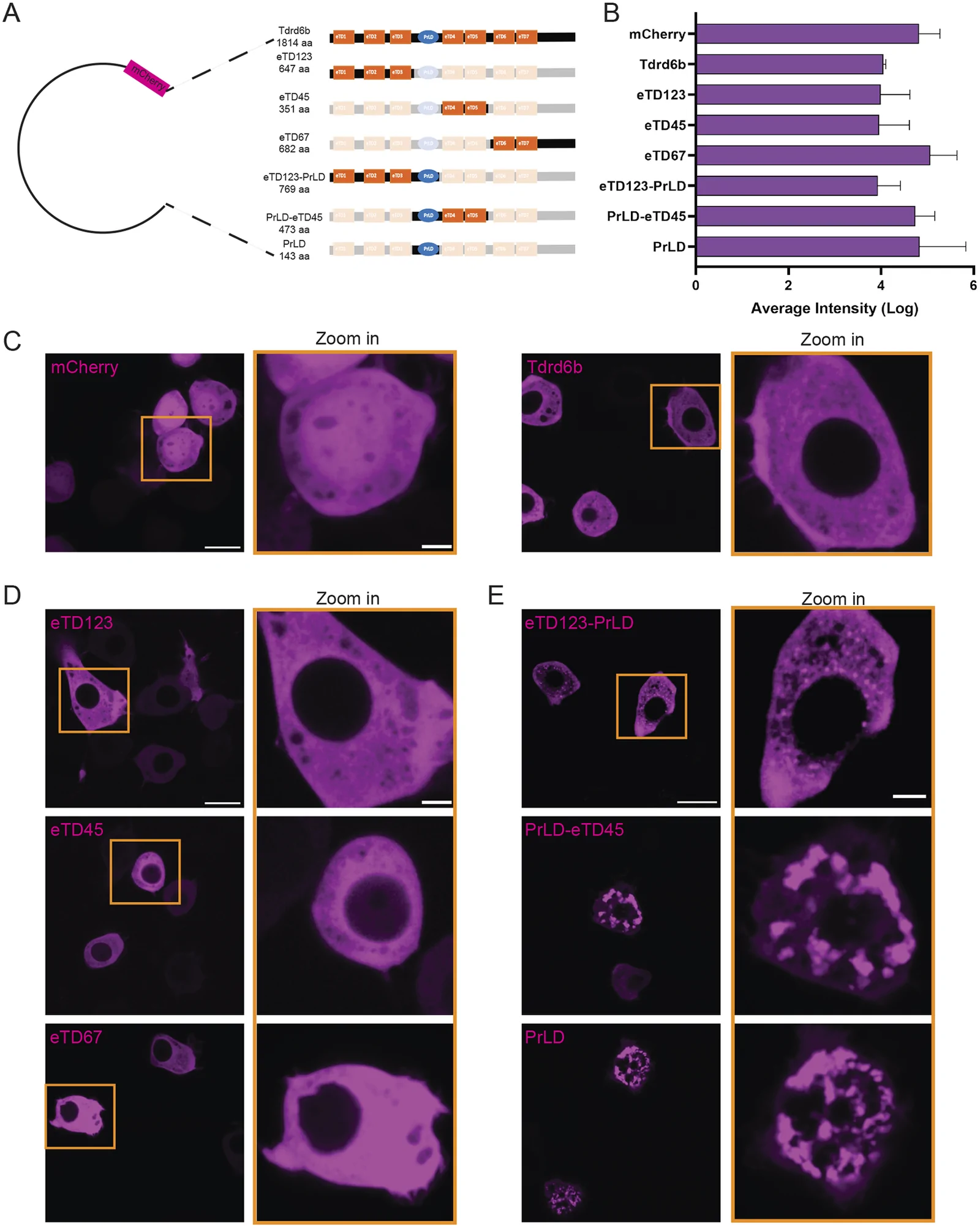
**Expression of Tdrd6b constructs in BmN4 cells.** (A) Schematic of plasmids expressing Tdrd6b constructs for BmN4 transfection. (B) Levels of expression measured for each tagged Tdrd6b construct based on the total fluorescence signal detected (for each construct, nine cells were measured). Error bars represent s.d. (C) Expression of mCherry compared to expression of mCherry-Tdrd6b. (D) Expression of Tdrd6b constructs that become diffuse in the cytoplasm of BmN4 cells. (E) Expression of Tdrd6b constructs that exhibit granular expression. Scale bars: 20 µm (C-E, left-hand panels); 5 µm (C-E, right-hand panels). Right-hand panels show the boxed areas at higher magnification. Transfections were carried out at least three times, with similar results.

Next, we aimed to study the aggregation behavior of Tdrd6b in more detail. For this, we chose to express different protein variants as N-terminally tagged mCherry (Shaner et al., 2004) fusion proteins in BmN4 cells (Fig. 6A). We chose these *Bombyx mori*-derived cells because of their germline character and because they are normally cultured at 28°C, which is the same temperature preferred for zebrafish breeding, making this system suitable for testing and analyzing zebrafish proteins (Roovers et al., 2018). The designed constructs reached similar levels of expression in the cytoplasm of BmN4 cells, as measured by the intensity of the mCherry fluorophore (Fig. 6B), and all the analyzed cells transfected with a particular construct showed similar subcellular distribution patterns of the mCherry signal (Fig. S7).

We started our analysis with transfection of the Tdrd6b full-length protein and observed that its signal was diffuse within the cytoplasm; no aggregation or particular foci of enrichment were observed for full-length Tdrd6b (Fig. 6C, Fig. S7), similar to what we observed before for Tdrd6a (Roovers et al., 2018). Its exclusion from the nucleus, in contrast to the mCherry control, likely reflects the size of the fusion protein. Constructs expressing combinations of solely eTDs behaved similarly to the full-length protein (Fig. 6D). In contrast, expression of the PrLD alone or in combination with adjacent sequences resulted in different subcellular localization patterns. When we expressed mCherry-PrLD or mCherry-PrLD-eTD45, we observed large cytoplasmic aggregates of irregular shape (Fig. 6E). By contrast, expression of mCherry-eTD123-PrLD revealed formation of smaller granules (Fig. 6E). These results indicate that the PrLD of Tdrd6b is both sufficient and necessary to confer self-interaction capabilities to Tdrd6b peptides in BmN4 cells. Interestingly, the three N-terminal eTDs, having incomplete aromatic cages, could modulate this self-aggregation behavior, whereas eTD4 and eTD5, which have intact aromatic cages, could not.

### Tdrd6b PrLD mediates Buc interaction

**Fig. 7.**
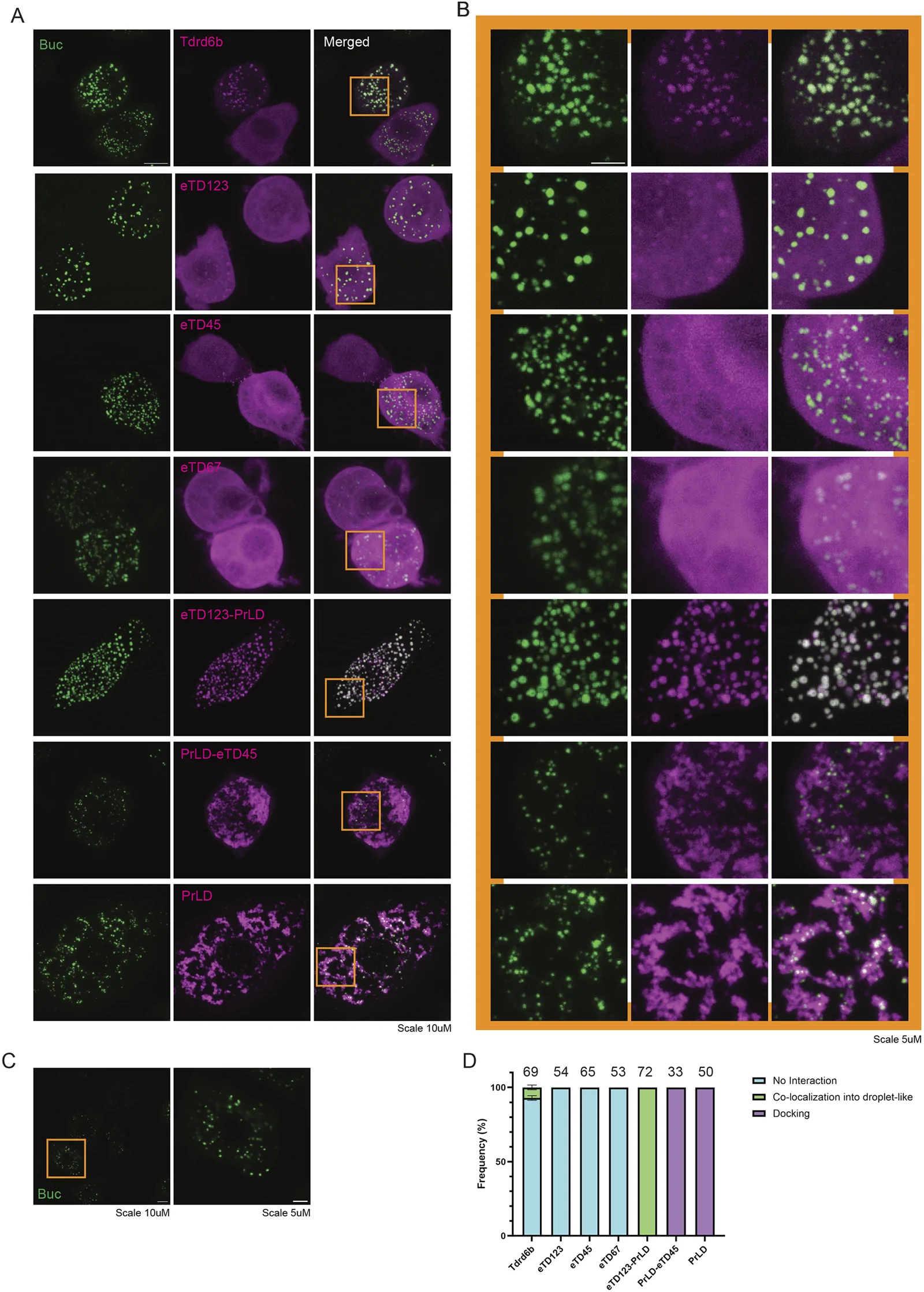
**Co-expression of Tdrd6b constructs together with Buc in BmN4 cells.** (A) Co-expression of BucGFP and different mCherry-tagged variants of Tdrd6b eTDs. Scale bar: 10 µm. (B) Higher magnification images of the boxed areas in A. Scale bar: 5 µm. (C) Expression of BucGFP alone. Scale bars: 10 µm (left); 5 µm (right). (D) Quantification of the frequency observed of the different kind of interactions of mCherry-tagged Tdrd6b variants with Buc condensates. Total number of cells analyzed, over three batches, is indicated by the numbers at the top of the columns. Confidence intervals reflect s.d. Transfections were carried out at least three times, with similar results.

Next, using this heterologous expression system, we wanted to test potential interaction between Tdrd6b and Buc. When Buc and full-length Tdrd6b were co-expressed, most cells (∼90%) showed Tdrd6b present throughout the cytoplasm, including the Buc granules, which formed independently of Tdrd6b (Fig. 7A-C). In ∼10% of cells, Tdrd6b was found to be enriched in Buc granules (Fig. 7A,B). Whether such enrichment of Tdrd6b in Buc granules correlates with changes in their size or other properties has not been addressed in this study. The difference between these two cell populations is unclear. It could relate to expression levels, but also to other aspects such as stage of the cell cycle or any other variable within this cell culture system. Nevertheless, these data show that Tdrd6b and Buc can interact. Furthermore, when expressing different combinations of eTDs of Tdrd6b, we did not detect strong interactions with Buc, as the latter formed round condensates whereas eTDs were mostly found to be localized diffusely in the cytoplasm (Fig. 7A). An exception was the eTD123 construct, which showed weak but detectable interactions with Buc condensates on occasion. By contrast, expression of constructs containing the PrLD showed interesting interactions with Buc. First, the TD123-PrLD protein always colocalized with Buc-positive round condensates in the cytoplasm (Fig. 7A,B). Second, mCherry-PrLD-TD45 formed large aggregates as before, and Buc droplets formed very close to, possibly on the surface of, these aggregates (Fig. 7A,B). Interestingly, Buc droplets appeared to be immobilized on the PrLD aggregates (Movie 3, Fig. S8). These results indicate that the PrLD of Tdrd6b interacts with Buc, and that the Tdrd6b eTDs 1-3 modulate the outcome of this interaction, likely by affecting the aggregation behavior of the Tdrd6b–PrLD complex itself. These experiments do not show that these interactions are direct.

## DISCUSSION

Mechanisms that rely on phase separation often require the involvement of proteins with multi-valency – the ability to interact with several substrates at the same time. Such multi-valency can stem from multiple folded domains, but also from intrinsically disordered regions or PrLDs (Banani et al., 2016, 2017; Shin and Brangwynne, 2017; Shorter and Lindquist, 2005). Interestingly, germline specification of different species has been characterized by the influence of phase-separated structures, raising the interest towards proteins and domains that regulate these processes in the light of germ cell specification (Boke et al., 2016; Bose et al., 2022; Brangwynne et al., 2009). In zebrafish, two multi-Tudor domain-containing proteins, Tdrd6a and Tdrd6b, have appeared as candidates to play a role in the dynamics of zebrafish Gp, both during oogenesis and embryogenesis. In this Discussion, we will point out the main implications and limitations of our study.

### Only Tdrd6a affects the Bb but does not affect oocyte maturation

We could not detect endogenous Tdrd6b expression. It remains unclear whether Tdrd6b is not expressed in oocytes, or maybe only at the very end of oogenesis, or whether cross-reactivity of our antibody prevented us from seeing the specific signal. Transgenically expressed Tdrd6b was found enriched in the nuage and diffuse throughout the cytoplasm. No specific enrichment of transgenic Tdrd6b within the Bb was observed. Furthermore, oocytes lacking Tdrd6b showed no Bb abnormalities, whereas Bbs were affected similarly in *tdrd6a* single and *tdrd6a;tdrd6b* double mutants. Many Bbs were aberrant in shape and *dazl* mRNA recruitment appeared impaired, similar to what we described before for *tdrd6a* single mutants (Roovers et al., 2018). Oocytes lacking Tdrd6a, or Tdrd6a and Tdrd6b, were able to develop into functional oocytes that could be fertilized, leading to viable offspring. Clearly, even if showing defects, the Bb, or at least its components, appears functional, as loss of the Bb-forming protein Buc has been reported to lead to dysfunctional (non-polarized) oocytes. More recently, however, we (Roovers et al., 2018) and others (Kobayashi et al., 2025 preprint) showed that in fact the Bb itself is not essential for axis formation. It seems more likely that the Buc protein itself is required, but not per se its aggregation into a large aggregate. Irrespective of precisely how Buc affects axis formation, the Tdrd6 proteins do not affect that function.

Given that both Tdrd6 paralogs are found in the perinuclear nuage in oocytes, they could have a role in the regulation of this structure and its function. However, here their role is likely modest, as disruption of perinuclear nuage in germ cells (by loss of Tdrd1) has been reported to lead to severe germ cell defects in zebrafish (Huang et al., 2011), while in oocytes mutant for *tdrd6a* and *tdrd6b* perinuclear nuage appears to form correctly, as judged from staining of Zili (Fig. S6).

### Tdrd6 proteins affect the Gp and PGC specification

After fertilization, both Tdrd6a and Tdrd6b are found enriched in the Gp droplets that aggregate at the cleavage furrows (Figs 1 and 2). When one of the two proteins is absent in embryos, Gp defects were minor in our analyses, which were based on Buc-eGFP protein localization and smFISH. However, in both scenarios, PGC numbers are reduced at the larval stage, indicating that the germline specification is impaired to some degree during early stages of development (Fig. 3). We hypothesize that this stems from defects in mRNA recruitment to the Gp.

Strikingly, in the absence of both Tdrd6 proteins, Gp is severely affected already at the 4-cell stage. Therefore, the embryo is unable to specify PGCs and mature into fertile fish. Although we did not reveal the molecular mechanisms with which Tdrd6a and Tdrd6b contribute to Gp stability, our previous study on Tdrd6a highlighted how Tdrd6a affects Buc mobility in aggregates, suggesting a direct role in the regulation of the phase-separation properties of Gp (Roovers et al., 2018). However, to understand the effects of Tdrd6a and Tdrd6b on Buc aggregation, studies with purified recombinant proteins will be required. We note, however, that the formation of both the Bb and the Gp is also likely affected by the presence of mRNPs that are abundantly found in these structures.

### Tdrd6b is prone to aggregation and interacts with Buc

Previous observations showed that Tdrd6a interacts with Buc through di-methylated arginine residues in the Buc C-terminal region (Roovers et al., 2018). Since these are known to be bound by eTD domains, the eTD domains of Tdrd6a, notably the two C-terminal eTDs of Tdrd6a, are likely involved in this interaction (Liu et al., 2010a,b). Notably, these have a complete aromatic cage (Fig. S2), which is involved in binding methylated arginine residues. In contrast, when we analyzed Tdrd6b domains in BmN4 cells, we observed that the main effect of the eTDs of Tdrd6b was on the aggregation of its PrLD (Fig. 6), triggering the formation of small cytoplasmic droplets, instead of the large, irregular aggregates formed by the PrLD alone. Additionally, Buc could merge with these granules, instead of the docking behavior observed in the PrLD aggregates in absence of these eTDs (Fig. 7). Notably, the first three eTDs of Tdrd6b are missing the aromatic cage and the key asparagine residue responsible for interaction with methylated substrates (Figs S2, S3) (Liu et al., 2010a,b). This would suggest that the first three eTDs have affinity for non-methylated residues within the Tdrd6b PrLD, thereby modulating its aggregation behavior. Such non-methylation-dependent interaction between an eTD and a substrate has been shown before (Webster et al., 2015).

Altogether, the differences in Buc interactions between Tdrd6a and Tdrd6b are intriguing, since *in vivo* they act (at least partially) redundantly. It seems likely that different modes of interactions may play a role in the interactions between Tdrd6 proteins and Buc, further increasing the multi-valency of the system. As multi-valency is crucial for phase-separation processes, one can easily imagine how the presence of both Tdrd6a and Tdrd6b helps the formation of the Bb and Gp. Finally, the many eTD domains of the Tdrd6 proteins likely also play a role in other interactions, such as with mRNPs. Evidence for this comes from previously published interaction studies of Tdrd6a, which revealed many mRNP-related factors (Roovers et al., 2018).

### Limitations of this study

We have thus far not been able to demonstrate whether the reported protein–protein interactions are direct or indirect. While it does not affect the conclusions of the study, it is of course relevant to resolve this in future studies. Interactions with RNA and mRNPs will also be relevant to study in that context.

Another limitation is that we could not rescue our mutant phenotypes. This implies that some aspect of the transgene is not fully functional. This could relate to the tag that was placed on Tdrd6b, to expression levels or timing, or to features we have not yet on our radar.

While our experiments could not detect clear interactions between the eTD domains of Tdrd6b on their own and Buc, these cannot be excluded. For instance, it is possible that the Tdrd6b–eTD interactions require the methylation and/or non-methylation of specific arginine residues, and the methylation status of the relevant arginines when expressed in BmN4 cells may not match the requirements.

The experiments in the BmN4 cells are qualitative, not quantitative. To be able to draw strong biochemical conclusions, studies with recombinant proteins will need to be performed.

## MATERIALS AND METHODS

### Materials and kits

Details of materials used can be found in Table S1.

### Zebrafish lines

Zebrafish strains were housed at the Institute of Molecular Biology in Mainz and bred and maintained under standard conditions (28°C water temperature and lighting conditions in cycles of 14:10 h light:dark). Larvae <5 days post-fertilization were kept in E3 medium (5 mM NaCl, 0.17 mM KCl, 0.33 mM CaCl_2_, 0.33 mM MgSO_4_) at 28°C. The zebrafish strains used for this work are listed in Table S1). Periodic out-crossing of each line was carried out by mating to zebrafish *wt* strains (AB or TUE). All experiments were conducted according to the European animal welfare law and approved and licensed by the ministry of Rhineland-Palatinate. All alleles we used are available at the EZRC (https://www.ezrc.kit.edu/). Note that Tdrd6b is sometimes named Tdrd6c in some of the lines, due to changes in nomenclature. Animals used for the data in this manuscript were below the age of 15 months. Genome editing was performed under licenses G 18-50055 (Tdrd6b-mKate transgene) and G 135-087_E10 (*tdrd6b*; then called *tdrd6c*). Alleles used can be found in Table S1.

### Cell culture

BmN4 cells were cultured at 27°C in IPL-41 (Gibco) medium supplemented with 10% fetal bovine serum (Gibco) and 0.5% Pen-Strep. For imaging, cells were grown in 8-well µ-slides (Ibidi, 80826). BmN4 cells were obtained from T. Kusakabe (National University Corporation Kyushu University, Agricultural Bioresource Sciences, Japan). Further details are available online (https://www.cellosaurus.org/CVCL_Z634). These cells were not authenticated and were not tested for *Mycoplasma*.

### Design and cloning of CRISPR gRNAs

CRISPR gRNAs were designed using CRISPRscan in the UCSC genome browser (Moreno-Mateos et al., 2015). Designed oligonucleotides were produced by Integrated DNA Technology and incorporated into pDR274 plasmid by restriction enzyme digestion followed by T4 ligation (NEB, M0202) (Hwang et al., 2013).

### Expression of CRISPR gRNAs

Plasmids containing gRNAs encoding sequences were linearized with DraI restriction enzyme (NEB, R0129L) and extracted from gel (QIAquick Gel Extraction Kit, 28704). Linearized sequences were used for RNA *in vitro* transcription (MEGAscript SP6 Transcription Kit, AM1330). Subsequently, expressed gRNAs were treated with Turbo DNase (Invitrogen, AM2239) for 30 min at 37°C and purified by precipitation and re-suspended in RNase-free water.

### RNA extraction and cDNA preparation

Ovaries from fish were homogenized in TRIzol Reagent (Thermo Fisher Scientific, 15596018) using a sterile pestle in a 1.5 ml sterile tubes. Samples were centrifuged at 4°C for 5 min at 12,000 ***g*** and the clear supernatant was transferred to a new tube. To each tube, 0.2 ml of chloroform were added. Tubes were vortexed for 20-30 s and then incubated for 2-3 min. Samples were centrifuged for 15 min at 4°C at 12,000 ***g***. The upper aqueous phase was transferred to new tubes and 0.5 ml of isopropanol were added. Samples were incubated 1 h at −80°C and then centrifuged for 10 min at 4°C at 16,000 ***g***. Supernatant was discarded and pellet re-suspended in 1 ml of 75% ethanol. Samples were vortexed and centrifuged for 5 min at 160,000 ***g*** at 4°C. Supernatant was discarded and samples were let dry. Pellets were re-suspended in 20 µl sterile water.

### RT-qPCR

For RT-qPCR, 8-month-old female gonads were used for the first experiment with *tdrd6a* knockout (ID 1179), *tdrd6b* knockout (ID 1178) and *wt* siblings (ID 1178). RNA was extracted using the QuickRNA Microprep kit (Zymo Research, R1051). There were four replicates for each genotype, comprising one ovary each. Four-cell-stage embryos came from crosses of *tdrd6a* knockout (ID 1179), *tdrd6b* knockout (ID 1178), double knockout fish (ID 1173) or *wt* siblings (ID 1179). For each genotype, four replicates were prepared using around 40 embryos per replicate. Embryos were directly lysed in Trizol (Invitrogen™, 15596026) and cleaned in a first step with PhaseLock gel tubes (5 PRIME, 2302810) by adding chloroform. This aqueous phase was then further purified with the Direct-zol RNA Miniprep kit (Zymo Research, R2051). cDNA was prepared using 1 µg of total RNA and the ProtoScript^®^ II First Strand cDNA Synthesis Kit (NEB, E6560), qPCR was performed either with Luna^®^ Universal qPCR Master Mix (NEB, M3003) or iTaq Universal SYBR Green Supermix (Bio-Rad, 1725120) on a QuantStudio 5 Real-Time-PCR-Systeme (Applied Biosystems). The ddCT− method was employed according to Livak and Schmittgen, without the logarithmic conversion. Primers were designed using PrimerBlast (https://www.ncbi.nlm.nih.gov/tools/primer-blast/), and can be found in Table S1.

### Gateway cloning of *tdrd6b-mKate2*

The plasmid containing the *tg(ziwi:tdrd6b-mKate2-tdrd6b3′UTR)* sequence was generated with the Tol2kit for multisite-Gateway cloning (Kwan et al., 2007). The *tdrd6b* ORF and the *tdrd6b-3′utr* were amplified from zebrafish ovarian cDNA. The *mKate2* sequence was amplified from existing plasmid in the lab and annealed to the *tdrd6b-3′utr* with incubation at 95°C for 5 min in a Thermocycler and gradual cool down. The *tdrd6b* ORF sequence and the *mKate2-tdrd6b-3′utr* sequence were cloned respectively into the pDonr221 and the p2r-P3 vectors using BP clonase reactions (Thermo Fisher Scientific, 11789020). The LR reaction (Thermo Fisher Scientific, 12538120) was performed using the two obtained plasmids from the BP reaction and the p5E_pziwi (containing the *ziwi* promoter) together with the destination vector tol2CG2 (Kwan et al., 2007).

### Injection of zebrafish zygotes

CRISPR gRNAs were diluted in 0.05% Phenol Red (Sigma-Aldrich, 143-74-8) to a final concentration between 100 and 500 ng/µl, together with 2 µM of Cas9 protein (NEB, M0646T). Tol2 plasmids were diluted in 0.05% Phenol Red to a final concentration of 100 ng/µl, together with Transposase-encoding mRNA (100 ng/µl). A volume between 2 and 5 nl (1-2.5 µg of each RNA or 200-500 ng of plasmid) was injected to *wt* zebrafish zygotes via glass needles capillaries (Harvard Apparatus, EC1 30-0038).

### Genotyping of zebrafish strains

DNA was extracted from caudal fin tissue amputated from anesthetized fish. Fins were incubated in Lysis Buffer (50 mM KCl, 2.5 mM MgCl_2_, 10 mM Tris, 0.5% NP40, 0.5% Tween 20, 0.01% w/v gelatine and 0.1 mg/ml Proteinase K, pH 8) for 1 h at 60°C, followed by proteinase K inactivation for 15 min at 95°C.

For genotyping purposes, in order to distinguish the *wt tdrd6b* allele from the *tdrd6b^mz69^* allele, the DNA was amplified by PCR using the following primers: AC_gen_9F (ATCCAGCCAGATCAGTTACGCATCCAGCCA) and AC_gen_9R (TGGTTTTTGCCATGGACCCC) to amplify the *wt tdrd6b* allele; AC_gen_12F (ACCAGTTGGTCAAGCCAGAA) and AC_gen_12R (AGTGAATGCTGAACCTTGGGT) to amplify the *tdrd6b^mz69^* allele.

In order to distinguish the *wt tdrd6b* allele from the *tdrd6b^mz87tg^* allele, the DNA was amplified by PCR using the following primers: AC_seq_6F (AACCAATGCAGGGAAGGACGA) and AC_seq_8R (ACCACAAGCTATCATCTCAATGT) to amplify the *wt tdrd6b* allele; AC_seq_6F (AACCAATGCAGGGAAGGACGA) and AC_568_gen6c_mKate2_Rv (GGGAGGTCTGCATTTTG) to amplify the *tdrd6b^mz87tg^* allele.

All PCR protocols were performed with TAQ polymerase provided in-house from the Protein Production Core Facility of IMB.

The sequence mutated *tdrd6b* locus in *mz69* allele was as follows (bold indicates deleted in *mz69*; underlined indicates premature stop codon resulting from the first 5 bp deletion):

GGAATCGTTAAGACCCGT**GGTTT**CAAGGAGACTTCAGAAGGCATGGAGTTTAGAGAGGTGAGAAATAGTTTTAGCACTACATCACAAGCACATGAGAGGACTTTAAAACCAGTTGGTCAAGCCAGAACAGAACAAAACTTCCAGTCTGCTACTGGATACAGCAATG**CAACTGGAAACAATAGAGCTTGGCCTAATGTTTTACAGCAACAAGTAATGGCTTATCCAAAGCTCACGGAACTTCCCTTGAGAACCATCGATGTGGGGTATGTTTCAGATGTCTATGTTTCACATTGTAATAGTCCATCAAATTTCTTTGTGCAATTGACAACTGATGAAGACGAAATATTTTCCCTTATTGAAAAACTCAATTGTACTTTGATAAGTGATTTACAAGTTGAACTAAATCTCTTGCAGTCAGATGATCTGGTGAAAGCTGAATATCCAGCCGATGGTGCCTATTATCGTGCTGTTGTAAAAAGCAAAAAAAATGGCATGGTTCAAGTGCAGTTTATTGACTTTGGGAATGAAGTGACACTCCTGCCCACTAAAATAAAGCAACTTGACAAGCAGTTCCTTAGCACTCCCAGGCTCAGTATTCAATGCATGCTTGAGGATGGAAAGAGACGAGGAAAAAGAGACTGGACCCAGGAGGAGATAATGACTTTCAAAGAGGCAGCAGGTGAAAATGGTGAAAAGAAAATTCAATGCAAGTTCATTCAAGAAGATGCAAATTGTTGGCTTGTAAGTTTAGAACACCAGGAAGGATGGTTTACTGTCAAATCAGATGCTCAAAGCGAATCCCAAGTACACTGCATTAAAAATACCCAGCCTTTAACTTTCAAGAAGCCAGCTGTAACTCCAGGACAAACTGTAGAGGCATATGCTTCATCTATCGTCGGACCAAATTACTTCTGGTGCCAGTTCAAAAACTCAGAGATTCTTGACAAAATCTCACTGCTTGCACAAGAAATTGGAAACAGTAGTCACACGAAAGCAATCCAGCCAGATCAGTTACGCCAAGGTGGAATTTGTTTGGCTCGCTTTTCTGACCAGCTCTGGTACAGAGCACAGGTTATCAATACCCATGAAAAGGTCTCTGTTCTTTTTATAGATTATGGAAATGAGTCTGAAATTGATTTTAACTCCATAAAGGCCCTGCCAACCAAGCTGCTGGAAAGCCCACCACAAGCCTTTCTATGTCAGTTGCAAGTTTTGGGGTCTGTAGAGGGCACATGGAGCGACAGTGCAGCTGATAAATTCTTTGAGATTCTAGTGGATGAACCTTTAAAAGTGACTGTTCAGAATATGGTGGTGACCCCTAACCCTACAATGTGTCCTCAGTTTAGTGTCCTTGTTGAATGCAAGGAGCTGATCGTTAATGATCTTATGAAAGATTTCTTGTCTGATCCCAAACCTCAAGTCTCTAGTAAGGGGTCCATGGCAAAAACCAATGCAGGGAAGGACGATTTTGGTGTAACTAACTCTGAAGCTGTCCAGAAAAATAAAACACCCAACCTTAACTCTAGTACAACACCATCAATTGATGATGTCAACAATTCAGCAGCATTGACCACAGGTGTAAACATGATGGATCCTGAATCAATGCAGTCTTTTCCTCGATCTGTAGATC**TTCCCCAACGTGTGATACA.

Primers can be found in Table S1.

### Gibson assembly cloning

Plasmids used for transfection of BmN4 cells and for protein expression were cloned using Gibson Assembly strategy (Gibson et al., 2009).

### PGC counts

At 24 hpf, eGFP-positive zebrafish embryos, obtained from vasa:eGFP-positive mothers, were dechorionated and fixed in 4% paraformaldehyde in PBS-Tw (PBS-Tween 0.1%). Then, samples were washed twice in PBS and placed in 8-well slides (Ibidi, 80826) and analyzed under the 10× objective of the Visitron VisiScope microscope and GFP-positive cells were counted manually.

### Recombinant protein production

pET vectors containing coding sequences for His14-TEVsite-DrBuc(fl), His6-MBP-TEVsite-DrTdrd6b(L634-K688) and His6-GST-3Csite-DrTdrd6b(L634-K688) were transformed into *Escherichia coli*: BioCat OverExpress C41(DE3) (Buc) and Agilent BL21-CodonPlus (DE3)-RIL (Tdrd6b). Cells were grown in 4 l of TB media (Formedium), containing 50 µg/ml kanamycin at 37°C at 140 rpm in an Infors Multitro 2 incubator until OD(600) of 1.2 (Buc), or 0.6 (Tdrd6b). Expression was induced by addition of 0.5 mM IPTG (neoFroxx) and further incubation at 37°C for 3 h (Buc), or 18°C overnight (Tdrd6b). Cells were harvested by centrifugation (4000 ***g***, 15 min, 4°C), re-suspended in ice-cold lysis buffer (Buc: 50 mM Tris-Cl, 6 M GdnHCl, 500 mM NaCl, 15 mM imidazole, 1 mM EDTA, 10 mM DTT, 0.1% Triton X-100, pH 8.0; Tdrd6b: 50 mM Tris-Cl, 500 mM NaCl, 15 mM imidazole, 1 mM MgCl_2_, 0.5 mM DTT, 1× Roche cOmplete EDTA-free protease inhibitor, 50 U/ml Sigma Benzonase, 0.1% Triton X-100, pH 8.0) and lysed by sonication at 4°C (Branson Sonifier 450, 9 mm tip, output 7, 20% duty cycle, 2×3 min). Cell lysates were cleared by centrifugation (40,000 ***g***, 30 min, 4°C). For Buc purification under denaturing conditions, cleared lysate was applied to 10 ml Ni-INDIGO bead slurry (Cube Biotech) that was pre-equilibrated with wash buffer (50 mM Tris-Cl, 4 M GdnHCl, 500 mM NaCl, 15 mM imidazole). After a 4 h rotating incubation at room temperature with the cleared lysate, beads were transferred into an Econo-Pac column (Bio-Rad) and washed with 100 ml wash buffer, before His14-TEV-Buc(fl) was eluted using 500 mM imidazole (pH 8.0, in wash buffer) for further purification. Lysates containing His6-MBP-TEV- and His6-GST-3C-tagged Tdrd6b(L634-K688) were applied to a HisTrap FF 5 ml column (Cytiva) at 4°C, using an automated chromatography system (Bio-Rad NGC Quest Plus). The column was washed with 20 CV (column volumes) of wash buffer (50 mM Tris-Cl, 500 mM NaCl, 15 mM imidazole, 5% glycerol). Proteins were eluted from the column by applying a linear gradient of 15-500 mM imidazole (in 50 mM Tris-Cl, 500 mM NaCl, 5% glycerol) over 10 CV and peak elution fractions were pooled for further purification. For the purification of untagged Tdrd6b, 50 mg of His6-MBP-TEV-Tdrd6b(L634-K688) were digested using 1 mg recombinant His6-TEV protease at 4°C overnight during a 1:100 (v/v) dialysis (50 mM Tris-Cl, 200 mM NaCl, 0.5 mM DTT, 5% glycerol), using SnakeSkin dialysis tubing with 3.5 kDa cut-off (Thermo Fisher Scientific). The dialyzed solution, containing untagged Tdrd6b(L634-K688), His6-MBP-TEV and His6-TEV protease, was supplemented with 15 mM imidazole (pH 8.0) and applied to a HisTrap FF 5 ml column. The flow-through containing untagged Tdrd6b(L634-K688) was collected for further purification. All three recombinant proteins were concentrated using Amicon Ultra-15 spin concentrators with 3 kDa cut-off (Merck Millipore) and subjected to size exclusion chromatography at 4°C (Cytiva Superdex 200 16/60 pg for Buc and GST-tagged Tdrd6b, Superdex 75 16/60 pg for untagged Tdrd6b) in 20 mM HEPES NaOH pH 7.4, 4 M GdnHCl for Buc and sterile PBS for untagged and GST-tagged Tdrd6b. Peak elution fractions of the proteins were analyzed by SDS-PAGE, pooled, spin-concentrated to 30 g/l (Buc), 2.4 g/l (GST-tagged Tdrd6b) and 0.67 g/l (untagged Tdrd6b), aliquoted, flash-frozen in liquid nitrogen and stored at −80°C. Untagged Tdrd6b(L634-K688) was oligomeric and eluted in the void volume of the Superdex 75 column.

### Polyclonal anti-Buc and anti-Tdrd6b antibody production

Polyclonal antibodies against recombinant His14-TEV-Buc(fl) and untagged Tdrd6b(L634-K688) were raised in guinea pig and rabbit, respectively, using Eurogentec's Speedy 28-day program. For each immunization round, 50 µg of Buc, dialyzed in PBS containing 4 M urea, and 200 µg of Tdrd6b in PBS were used. For antibody purification from serum, 1.5 mg of His14-TEV-Buc(fl) and 1.2 mg of His6-GST-3C- Tdrd6b(L634-K688) were diluted with 1 ml coupling buffer (50 mM Tris, 5 mM EDTA, pH 8.5, additional 5 M GdnHCl for Buc) and incubated with 1 ml SulfoLink bead slurry (Thermo Fisher Scientific), pre-equilibrated in coupling buffer. The proteins were covalently coupled to beads by incubation at room temperature for 2 h in a Poly-Prep column (Bio-Rad) while rotating. After washing with coupling buffer, the remaining iodoacetyl groups were blocked with 5 ml quenching buffer (50 mM L-cysteine in coupling buffer) for 30 min. The resin was subsequently washed with 10 ml coupling buffer containing 1 M NaCl, followed by 20 ml PBS. For antibody purification, 2 ml guinea pig serum (Buc) and 5 ml rabbit serum (Tdrd6b) was diluted 1:2 with PBS, filtered through a 0.45 μm filter and incubated with Buc- or Tdrd6b-coupled SulfoLink resin in a Poly-Prep column overnight at 4°C while rotating. Resins were washed with 10 ml PBS containing 0.1% Triton X-100 followed by 20 ml PBS. Antibodies were eluted with 6×0.5 ml low pH buffer (200 mM glycine-HCl, 150 mM NaCl, pH 2.3) and immediately neutralized with 0.1 ml of 1 M Tris-Cl pH 8.5. Eluted fractions were analyzed by SDS-PAGE and rebuffered into storage buffer (PBS, 10% glycerol, 0.05% NaN_3_) using PD-10 columns (Cytiva). Antibodies were spin-concentrated to 1 g/l, aliquoted, flash-frozen in liquid nitrogen and stored at −80°C.

### Whole-mount immunohistochemistry

Zebrafish samples (ovaries, embryos or larvae) were stored in methanol at −20°C overnight and were rehydrated in either PBS-Tw/methanol series (50%, 75%, 87.5% and 100%) (embryos and larvae) or PBS-Triton 0.3% methanol series (oocytes). Two additional washing steps in 1% Triton X-100 in PBS were used for larvae and in 0.3% PBS Triton for ovaries. Antigen retrieval was performed with incubation of the samples in 150 mM Tris (pH 9) solution at room temperature for 5 min and then at 70°C for 15 min. Samples were then blocked in Blocking Solution [8% bovine serum albumin (BSA) in PBS-Tw] for 90 min at room temperature with gentle agitation. After blocking, samples were incubated overnight at 4°C with diluted primary antibodies (anti-Tdrd6a 1:500, anti-Tdrd6b 1:100, anti-Zili 1:500, anti-Ziwi 1:100 and anti-Buc 1:100) in Blocking Solution and with gentle agitation. Samples were washed six times (30 min each wash) in PBS-Tw and then incubated in Blocking Solution with 1:500 dilution of secondary antibodies. Samples were washed in PBS and gently transferred to Ibidi slides (8-well slides). Confocal *z*-stacks were acquired, and the resulting three-dimensional data sets were converted into two-dimensional images using the Maximum Intensity Z-Projection function in Fiji (ImageJ). See Table S1 for antibody details.

### Whole-mount double smFISH and IHC

smFISH staining was performed as described previously (Roovers et al., 2018). Briefly, embryos were collected and fixed as described above. Following rehydration, the embryos were incubated overnight with 1:100 anti-Buc and 1:100 Quasar-labeled *vasa* smFISH probes (Stellaris custom design; see Roovers et al., 2018 for probe sequences) stocks (12.5 mM in TE buffer) in hybridization buffer [10% dextran sulfate, 10% formamide, 1 mg/ml tRNA, 0.02% BSA, 2 mM vanadyl-ribonucleoside complex (NEB S1402S) in 2×SSC]. The following day, embryos were washed in 10% formamide in 2×SSC and incubated with 1:500 anti-guinea pig 405S and mounted in Ibidi mounting medium. Confocal *z*-stacks were acquired, and the resulting three-dimensional data sets were converted into two-dimensional images using the Maximum Intensity Z-Projection function in Fiji (ImageJ).

### Live imaging of zebrafish embryos

Zebrafish embryos were collected in Petri dishes containing E3 solution manually de-chorionated. With glass pipettes, embryos at the 1-cell stage were gently transferred to 8-well slides (Ibidi, 80826). Gently, excess E3 was removed keeping the samples under the level of the surface of the solution and PBS containing ∼0.5% low gelling agarose was added one drop at the time. Samples were imaged in the VisiScope system (Leica) with a 20× objective.

### smFISH

smFISH was performed on fixed zebrafish oocytes which had been stored overnight at −20°C, in 100% methanol. Oocytes were rehydrated in a 0.3% Triton X-100 PBS/methanol series (50%, 75%, 87.5% and 100%). Antigen retrieval was carried out as described in the ‘Whole-mount immunohistochemistry’ section. Samples were then blocked in hybridization buffer (10% dextran sulfate, 10% formamide, 1 mg/ml tRNA 1 mg/ml 2× SSC, 0.02% BSA, 2 mM Vanadyl-ribonucleoside complex) for 1 h at 30°C. They were then probed with a custom Stellaris probe set targeting *dazl* (1:100) and a homemade antibody against Buc (1:100) overnight at 30°C. Following this, samples were washed in wash buffer (10% formamide, 2×SSC) for 15 min to wash off primary antibody, then a second wash step with added secondary antibody (1:500) for 30 min. Finally, two wash steps of 15 min each were carried out. All wash steps were performed at 30°C. Samples were mounted in antifade medium and imaged on a Visitron confocal microscope.

### BmN4 cell transfection and imaging

BmN4 cells grown in 25 ml flasks were re-suspended and counted automatically (Bio-Rad, TC20 Automated Cell Counter). Approximately 80,000 cells were seeded in each well of an 8-well slide (Ibidi, 80826) in a volume of 300 µl IPL-41 medium (Gibco) supplemented with fetal bovine serum (Gibco) and 0.5% Pen-Strep. The next day, 1 µl of each plasmid (from stock of 100 ng/µl) was in solution with 0.5 µl X-tremeGENE_HP (Roche) and 28.5 µl IPL-41 (Gibco) medium supplemented with 10% fetal bovine serum (Gibco) for 20-30 min prior to transfection. Transfected cells were imaged 2 days after transfection at the Leica TCS SP5 Confocal microscope with a 63× objective.

### Quantification and statistical analysis

Quantifications of microscopy images were carried out either manually or with pipelines within Arivis Software (Zeiss).

A two-sided Wilcoxon test was used to calculate significant differences between populations of maternal mutants in the following experiments: measurement of PGC and male abundance. Tests were corrected for multiple testing within the respective data set. No data were excluded. We did not pre-specify effect sizes.

Expression patterns in BmN4 cells were quantified by counting cells with the indicated expression characteristics while performing the microscopy. Images were taken from cells that are represented in the figures.

No randomization of samples was applied in these studies and investigators were aware of sample groupings during analysis.

### Source data

Source data related to Figs 3C,D, 4G, 5C, 6B and 7D can be found in Table S2.

## Supplementary Material

10.1242/develop.204545_sup1 Supplementary information

Table S1. Table with information on materials used in this study.

Table S2. Source data to Figs 3C, 3D, 4G, 5C, 6B, 7D.
