# Supplementary material for: Tdrd6a and Tdrd6b are required together for germ plasm formation
Source: Development. 2026 Mar 27;153(6):dev204545. doi: 10.1242/dev.204545 (PMC13495786; doi:10.1242/dev.204545)
Supplement: Supplementary file 1 [file develop-153-204545-s1.pdf]

A

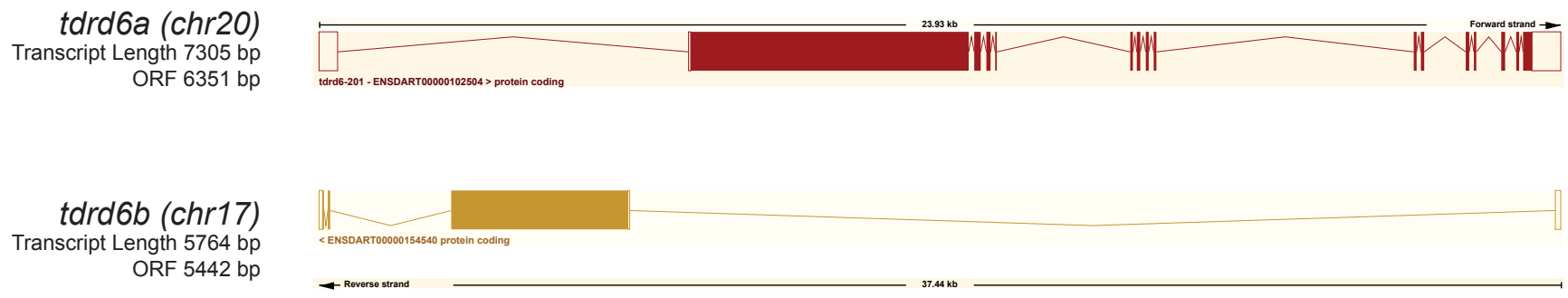

B

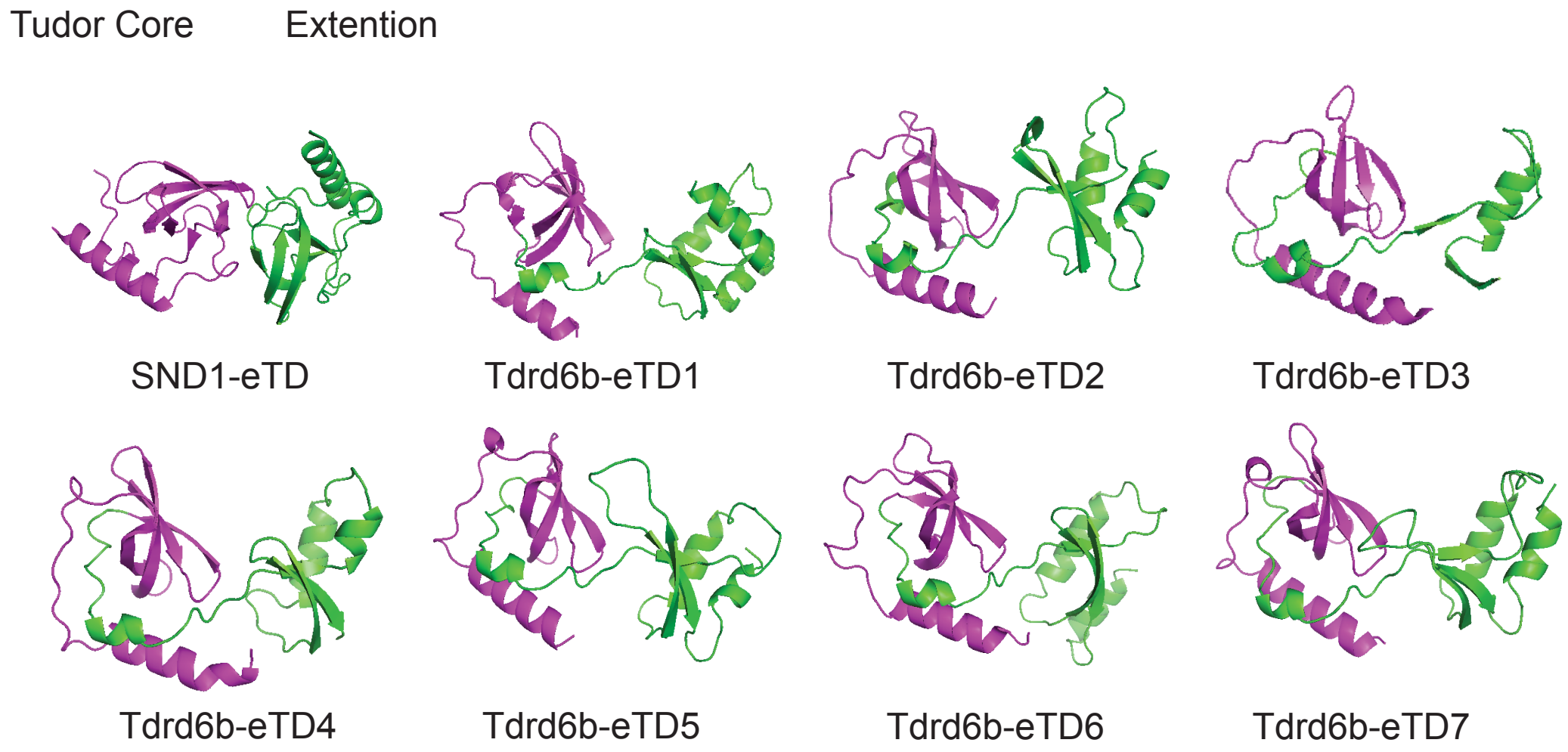

C

```
SND-eTD        ---SFKVGDYARATYV-DGVDYEGAVVSINEEKGTCVLRYLGYQNRSTTHVNELRERTI-    55
Tdrd6b-eTD1    HAFEGKPGEICLV--YEDRTWHR-ARIISE-TEYEYNVFLIDDGKSLWTTSEVLTKCPHN   115
Tdrd6b-eTD2    IPVPLCDGSPCATKGS-DGKWYR-SVLQQNDISDVVKVFHVDYGIGDFVKLSNIRPLSAR   345
Tdrd6b-eTD3    AKFSISPKMMCLAKFS-DGQWHR-GQI-ES-VHPKILVQFVDYGDLVVVDKSDILPFPPN   613
Tdrd6b-eTD4    -LNLLQSDDLVKAEYPADGAYYR-AVVKSK-KNGMVQVQFIDFGNEVTLLPTKIKQLDKQ   886
Tdrd6b-eTD5    QPDQLRQGGICLARFS-DQLWYR-AQVINT--HEKVSVLFIDYGNESEIDFNSIKALPTK  1085
Tdrd6b-eTD6    HGLSVQQGSLVKAMFPEDDSWYR-AVVKCAPENDMVFVEFIDFGNEAMVSSLKICRLDEH  1352
Tdrd6b-eTD7    LLDNLDSGSPCLARFCDDQMWYR-AQVIKK-CTNTVSVLFVDFGNESEASEGSVKALPCD  1557
```

Fig. S1. TDs of Tdrd6b are extended TDs.

(A) View of the zebrafish *tdrd6 loci* as annotated from Ensembl database. (B) AlphaFold predictions of the 7 eTDs of Tdrd6b compared to the eTD of the SND-1 protein; in magenta the Tudor core and in green the extended domain. (C) Alignment of the amino-acid sequences of the 7 eTDs of Tdrd6b and the eTD of SND-1; highlighted: in magenta are residues contributing to the aromatic-cage of canonical eTDs; in green the Asparagine residue that also interacts with methylated-Arginines of a substrate.

Development • Supplementary information

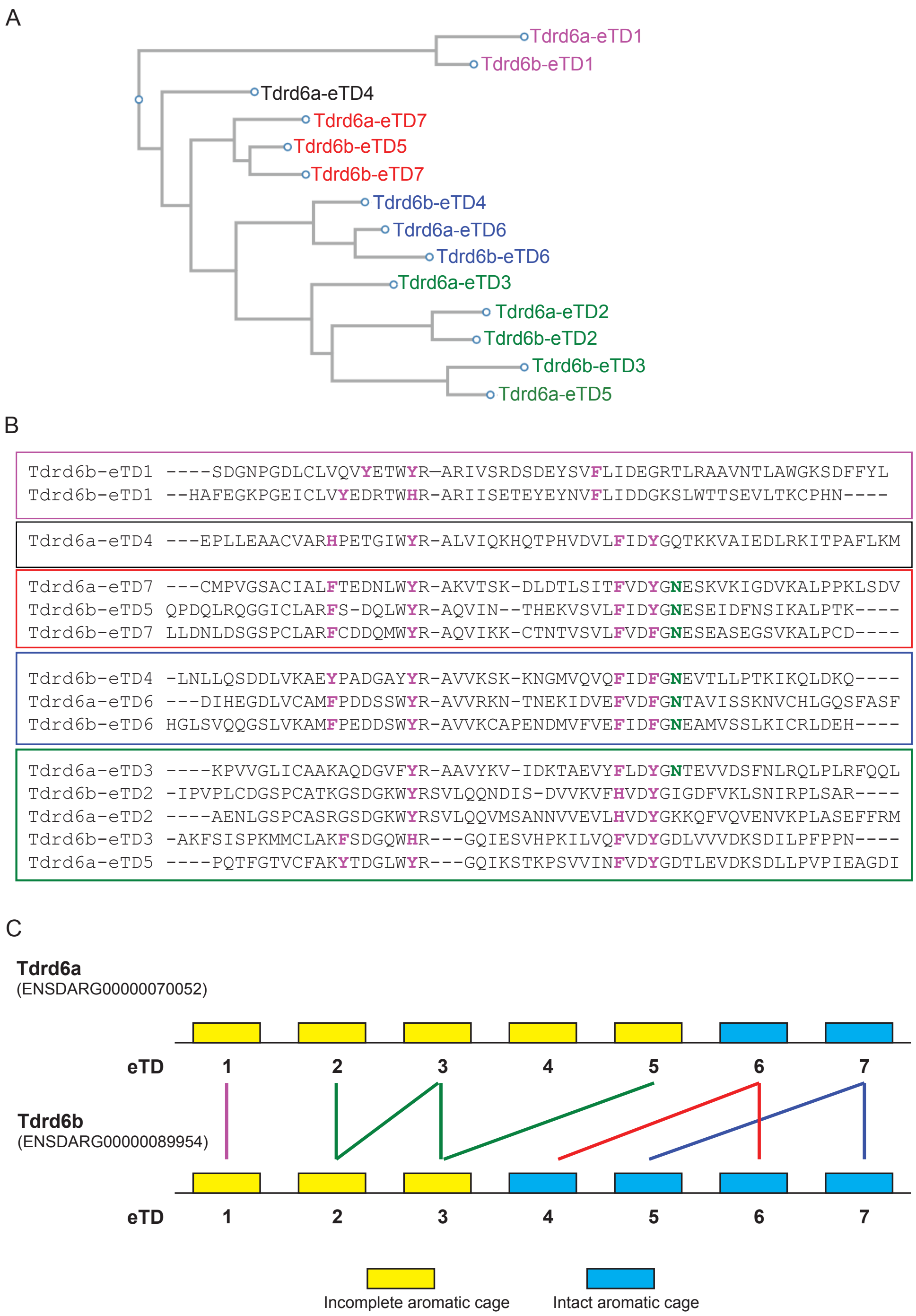

Fig. S2. Comparison of eTDs of Tdrd6a and Tdrd6b.

(A) Cladogram of eTDs from Tdrd6a and Tdrd6b, highlighting 5 groups (color coded) of eTDs. (B) Sequence alignment of the eTDs of Tdrd6a and Tdrd6b, used to generate panel A in CLUSTALW. Colored boxes, matching panel A, highlight the different groups. Residues in red are the ones supposed to contribute to the formation of the aromatic cage, in green the Asparagine residue that should bind methylated-arginine residues. (C) Schematic representation of Tdrd6a and Tdrd6b protein structure with their eTDs. Colored lines refer to the cladogram and indicate similarity between different eTDs.

A

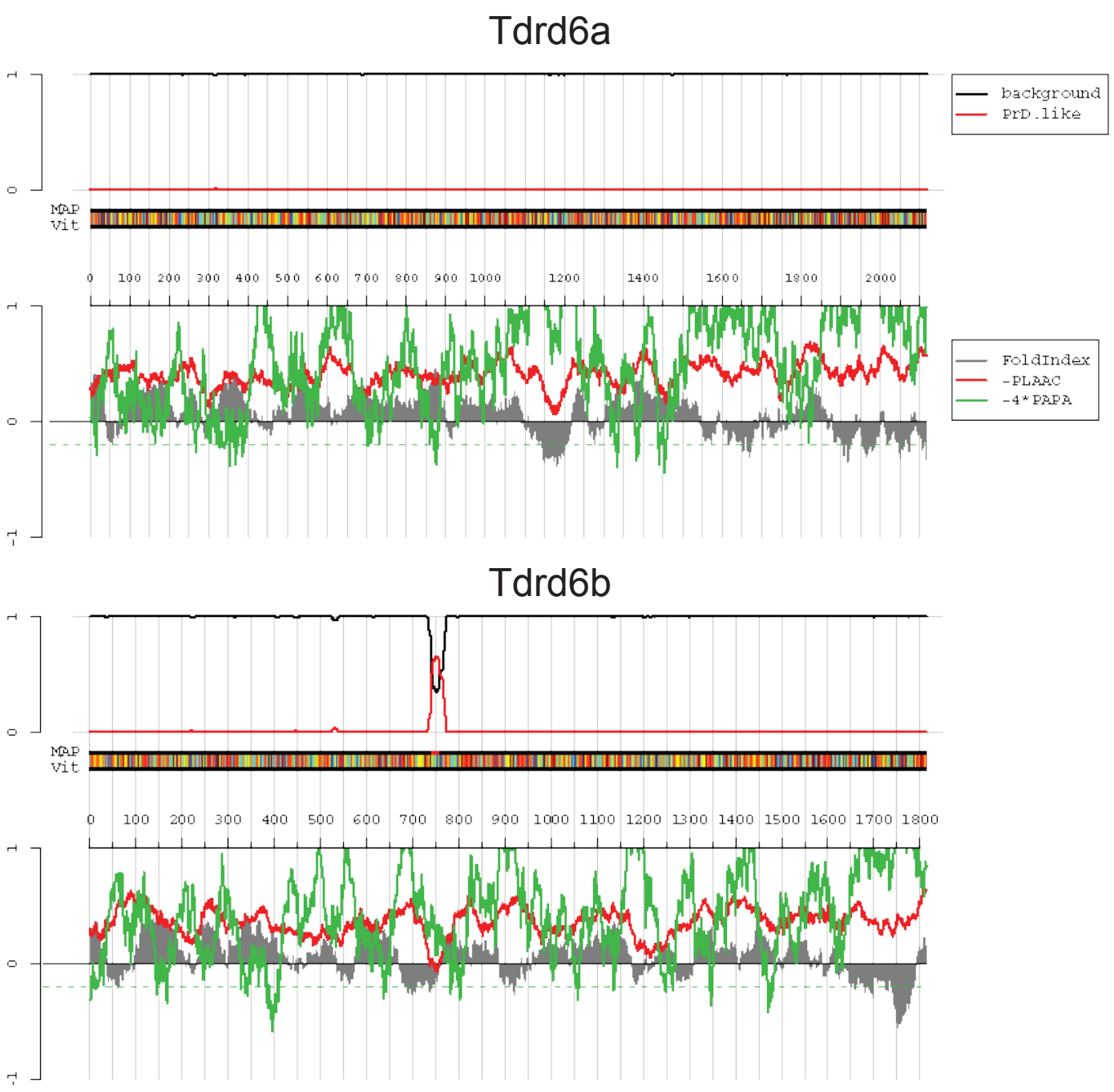

B

```
Tdrd6a      VRDRIIDLHVQDKQQDTHIVQLVDPSL--------------DG-EKDVSKLLCNAGFAVS   659
Tdrd6b      ATDCMFNVVVKLNSSGKLSVEMYDDKTNLNLKIKDLWSKTKRNESINTKGIVKTRGFKET   707
            . * :::: *: :....    *:: * .              . . :.. :: . **  :

Tdrd6a      EKSIV------DYSATRSCGLKTTHASGV------FL--------TGTQPQTPCS-SSVV   698
Tdrd6b      SEGMEFREVRNSFSTTSQAHERTLKPVGQARTEQNFQSATGYSNATGNNRAWPNVLQQQV   767
            .:.:       .:*:* ..    :* :    *   *         **.:   *   .. *

Tdrd6a      MD--SASAFKEYLFPIGSSLEVTVSYIENPNDFWCQKARNAACLEVLMQDIQRF-YSH--   753
Tdrd6b      MAYPKLTELPLRTIDVGYVSDVYVSHCNSPSNFFVQLTTDEDEIFSLIEKLNCTLISDLQ   827
            *   . : :    : :*   :* **: :.*.:*: * : :   :  *::.::    *.
```

Fig. S3. Tdrd6b predicted Prion-like domain.

(A) PLAAC prediction of Prion-like domains in Tdrd6a (Top) and in Tdrd6b (Bottom)(Lancaster et al., 2014)Click or tap here to enter text.. (B) Alignment of a part of Tdrd6a and Tdrd6b highlighting, in blue, that Tdrd6b predicted Prion-like domain is a unique sequence between the 2 proteins.

Development • Supplementary information

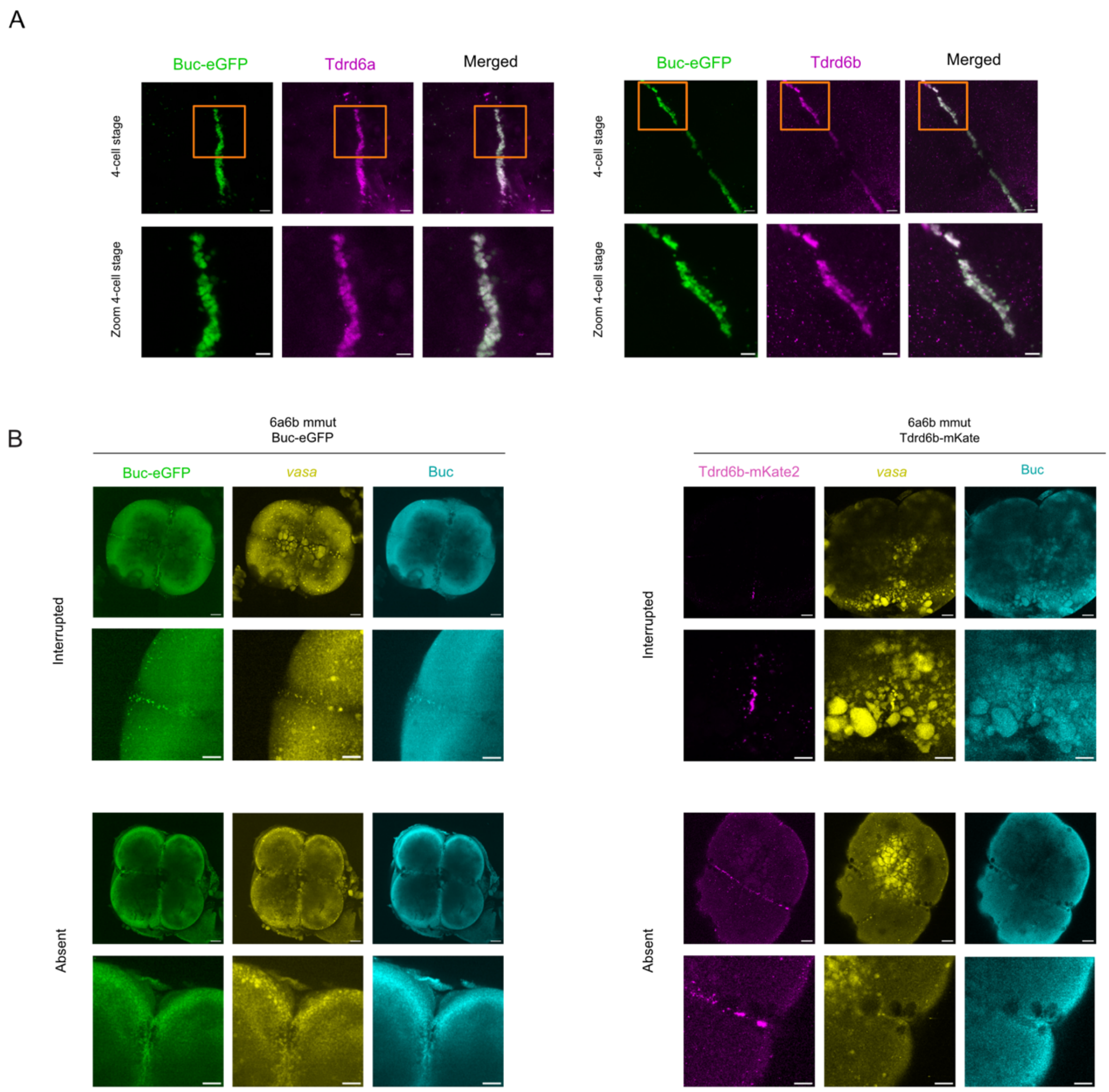

Fig. S4. Colocalisation of Tdrd6a and Tdrd6b with Buc-eGFP and subsequent loss of both mRNA and protein in 6a6b mmut embryos.

(A) IF staining of 4-cell stage embryos expressing Buc-eGFP (green) together with either Tdrd6a or Tdrd6b (magenta). Lower panels show zoomed-in views. Scale bars: 50 µm and 25 µm for overview and zoomed-in images respectively. (B) IF combined with smFISH for *vasa* mRNA in 6a6b mutant embryos with wither Buc-eGFP or Tdrd6b-mKate. Buc-eGFP IF (green), Tdrd6b-mKate2 (magenta), vasa (yellow) and Buc (cyan). Bottom panels show corresponding higher-magnification views. Scale bars: 50 µm for overview and 25 µm for zoomed-in images. The displayed stainings were observed in at least 2 independent experiments.

Development • Supplementary information

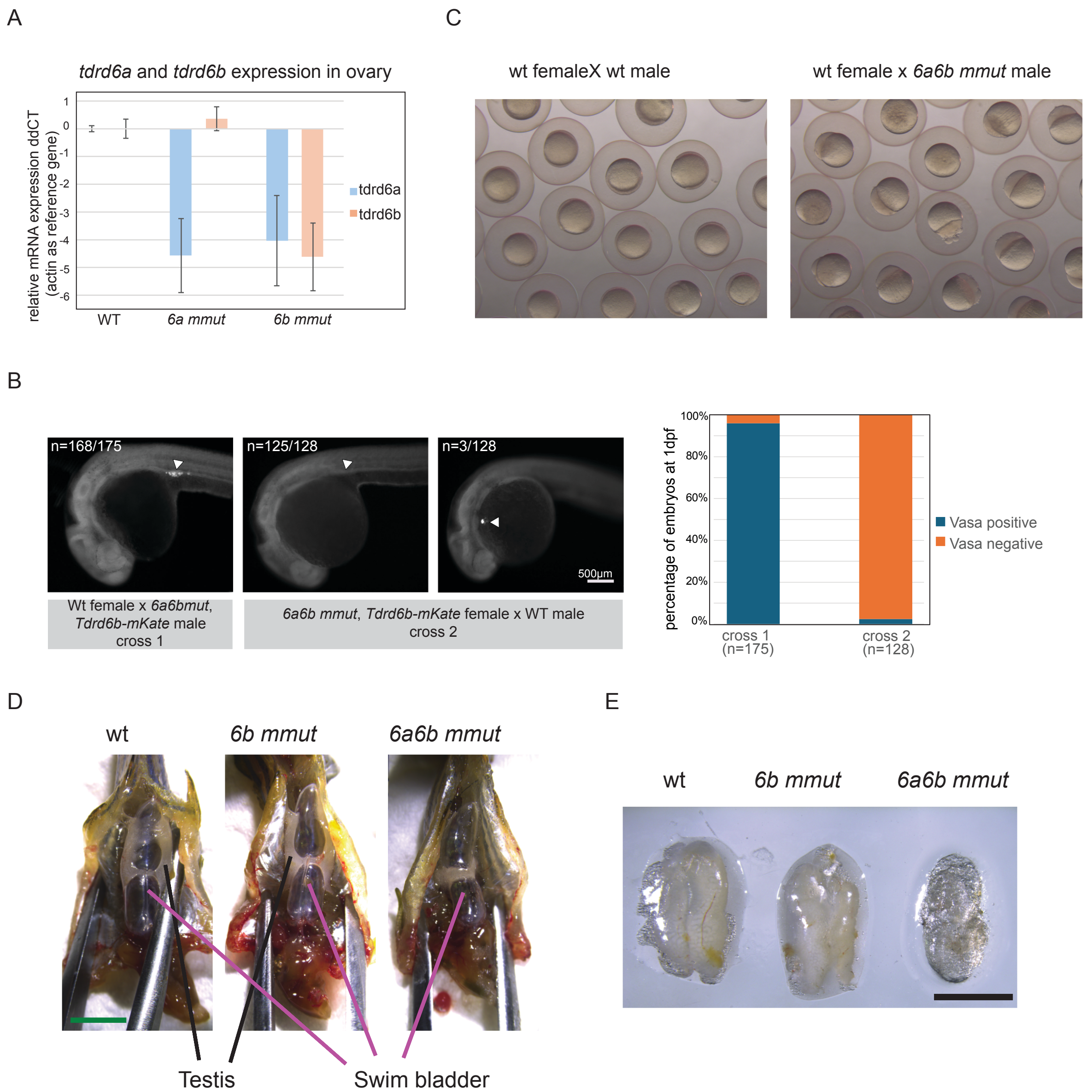

Fig. S5. Maternal Tdrd6a and Tdrd6b are required for normal gonad development.

(A) RT-qPCR analysis of *tdrd6a* and *tdrd6b* transcripts in ovaries of the indicated genotype. This experiment was performed once, with four biological replicates. (B) Detection of PGCs in 24 hour old embryos obtained from the indicated crosses. The area where PGCs should be located is indicated with a white arrowhead. In cross 2, we occasionally did detect a PGC but it had not properly migrated to the primordial gonad region (outlined arrowhead). (C) Collection of offspring from wt fathers (left) and from *6a6b mmut* fathers (right); wt eggs are fertilized and develop correctly when mating occurs with *wt* fathers while they appear unfertilized when collected from mating of *6a6b mmut* males. (D) Dissection of *6a6b mmut* zebrafish reveals that their gonads are not developed, in contrast to *wt* and *6b mmut* fish (scale bar = 5 mm). Only a small rudimentary part of the gonad structure can be seen crossing the swimming bladder. (E) Isolation of gonads from *wt, 6b mmut* and *6a6b mmut* further highlights that gonads fail to mature in *6a6b mmut* (scale bar = 3 mm). In wt and *6b mmut* samples the fat patch was overlain by milky-white tissue, which is the testis. In the *6a6a mmut* sample only the fat patch was visible, and testis tissue could not be clearly distinguished. Results from panels B-E stem from one experiment, but represent phenotypes from several individuals.

Development • Supplementary information

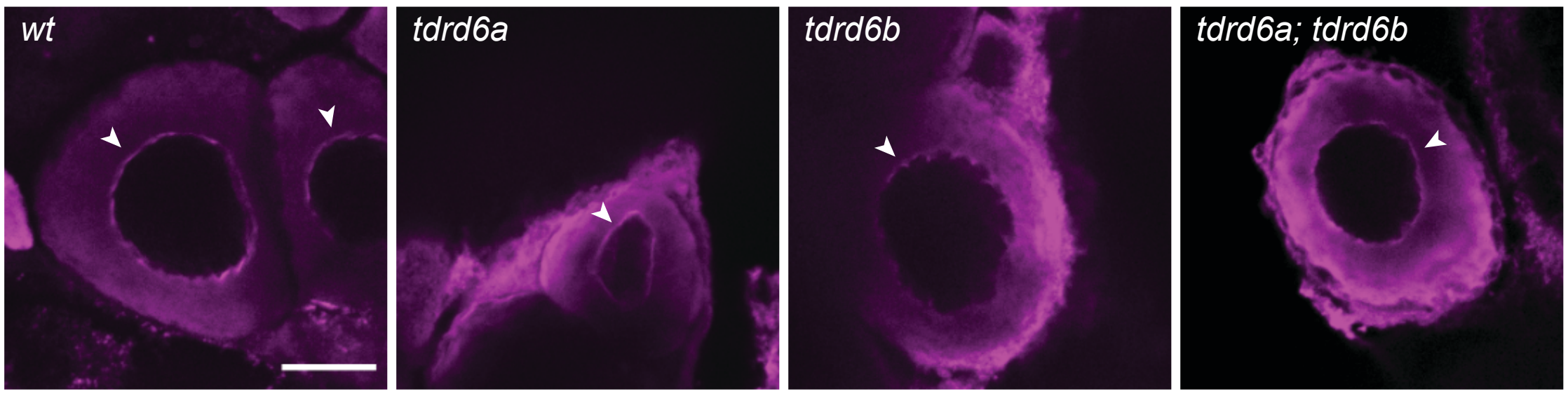

Fig. S6. The *nuage* appears correctly formed in *tdrd6a* and *tdrd6b* mutant oocytes. Perinuclear *nuage* (magenta, arrowheads) stained with antibody against Zili in *wt* and mutant oocytes. Scale bar = 50 µm. This experiment was done once.

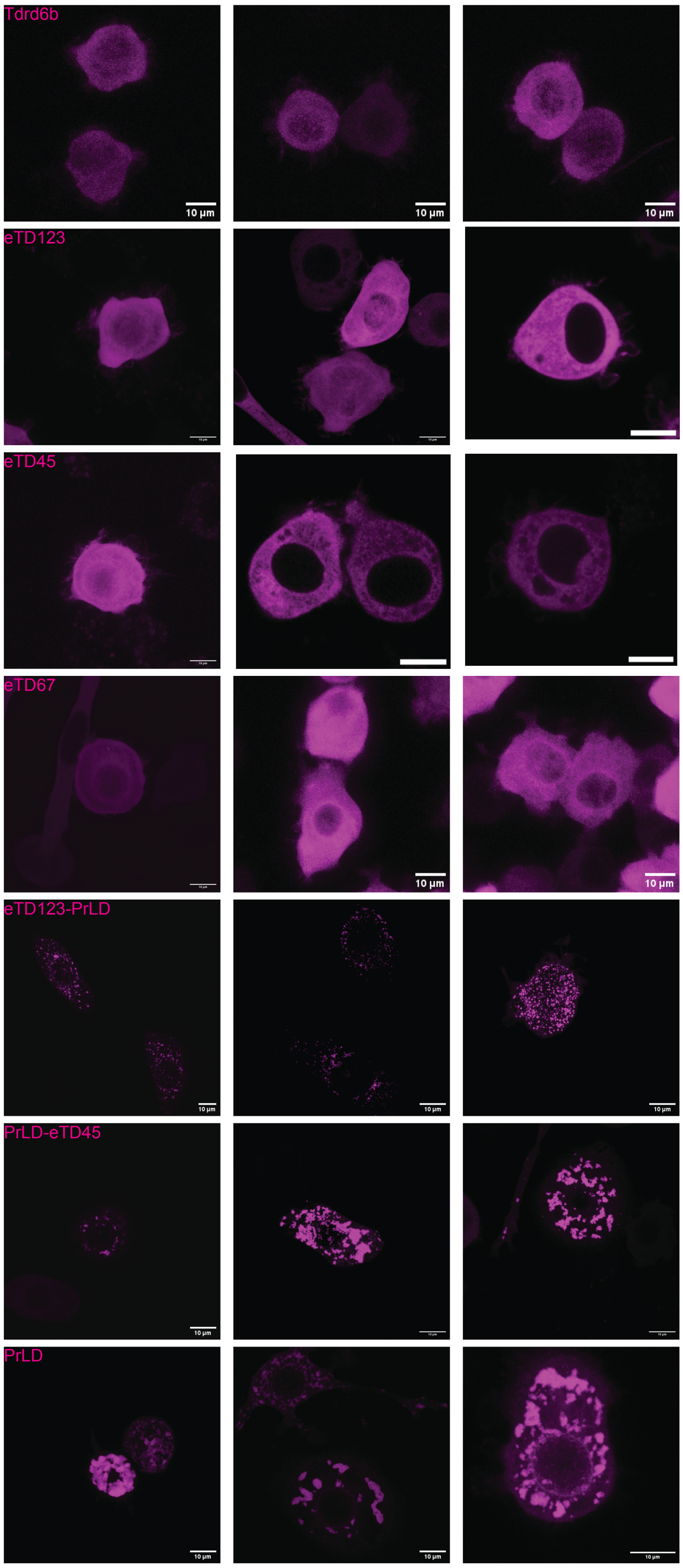

Fig. S7. Expression of Tdrd6b constructs in BmN4 cells.

Additional representative images of cells expressing the indicated constructs. Scale bars: 10 µm.

Development • Supplementary information

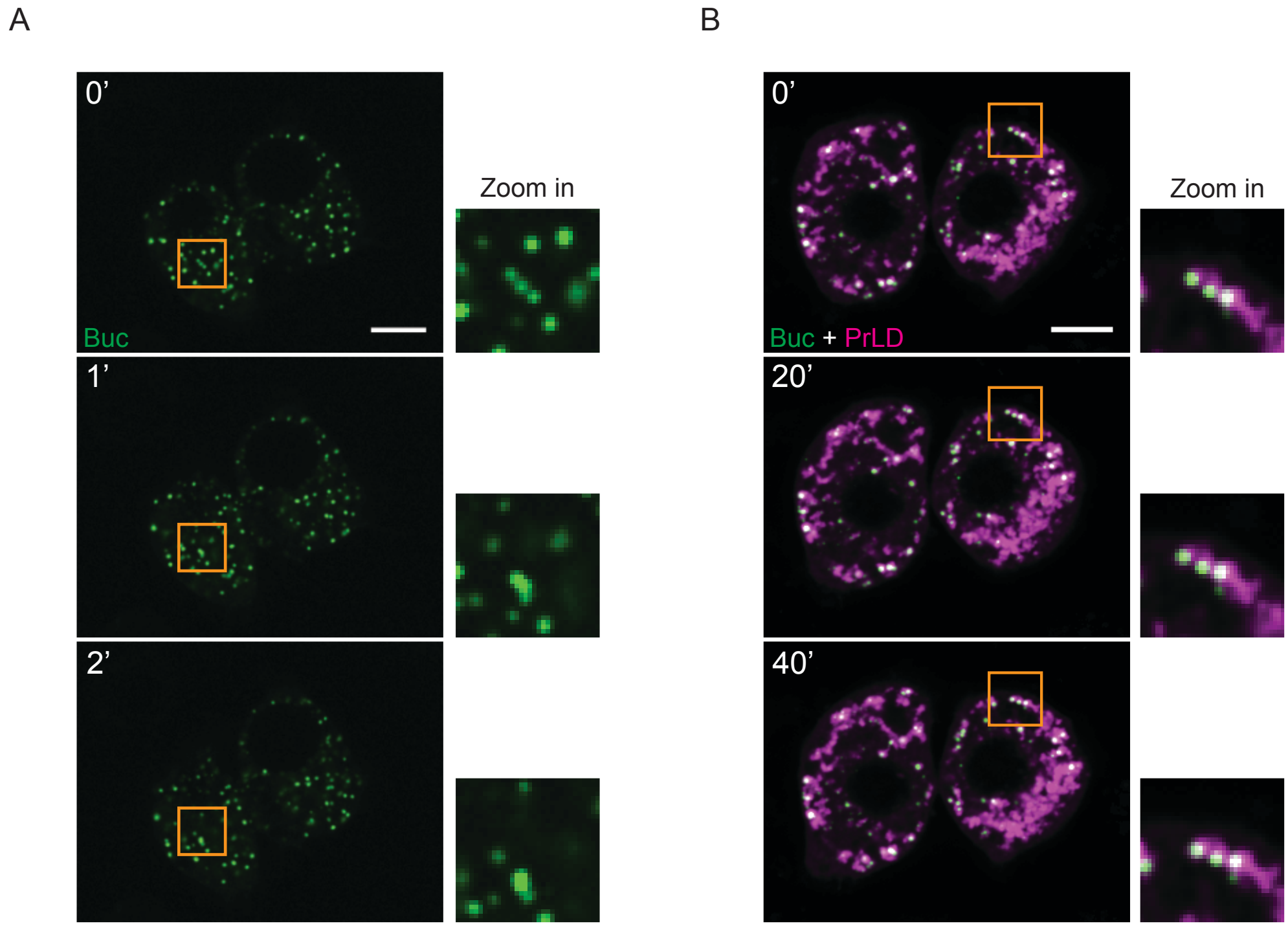

Fig. S8. BucGFP mobility in BmN4 cells.

(A) Buc-eGFP expression observed in BmN4 cells over 2 minutes. The displayed images are stills from Movie S2. (B) Buc-eGFP and mCherry-PrLD co-expression observed over 40 minutes in BmN4 cells. The displayed images are still from Movie S3. (C) Orange boxes indicate areas of zoom in. Scale bars = 10 µm. This experiment was performed once.

Table S1. Table with information on materials used in this study.

Available for download at
https://journals.biologists.com/dev/article-lookup/doi/10.1242/dev.204545#supplementary-data

Table S2. Source data to Figs 3C, 3D, 4G, 5C, 6B, 7D.

Available for download at
https://journals.biologists.com/dev/article-lookup/doi/10.1242/dev.204545#supplementary-data

Development • Supplementary information

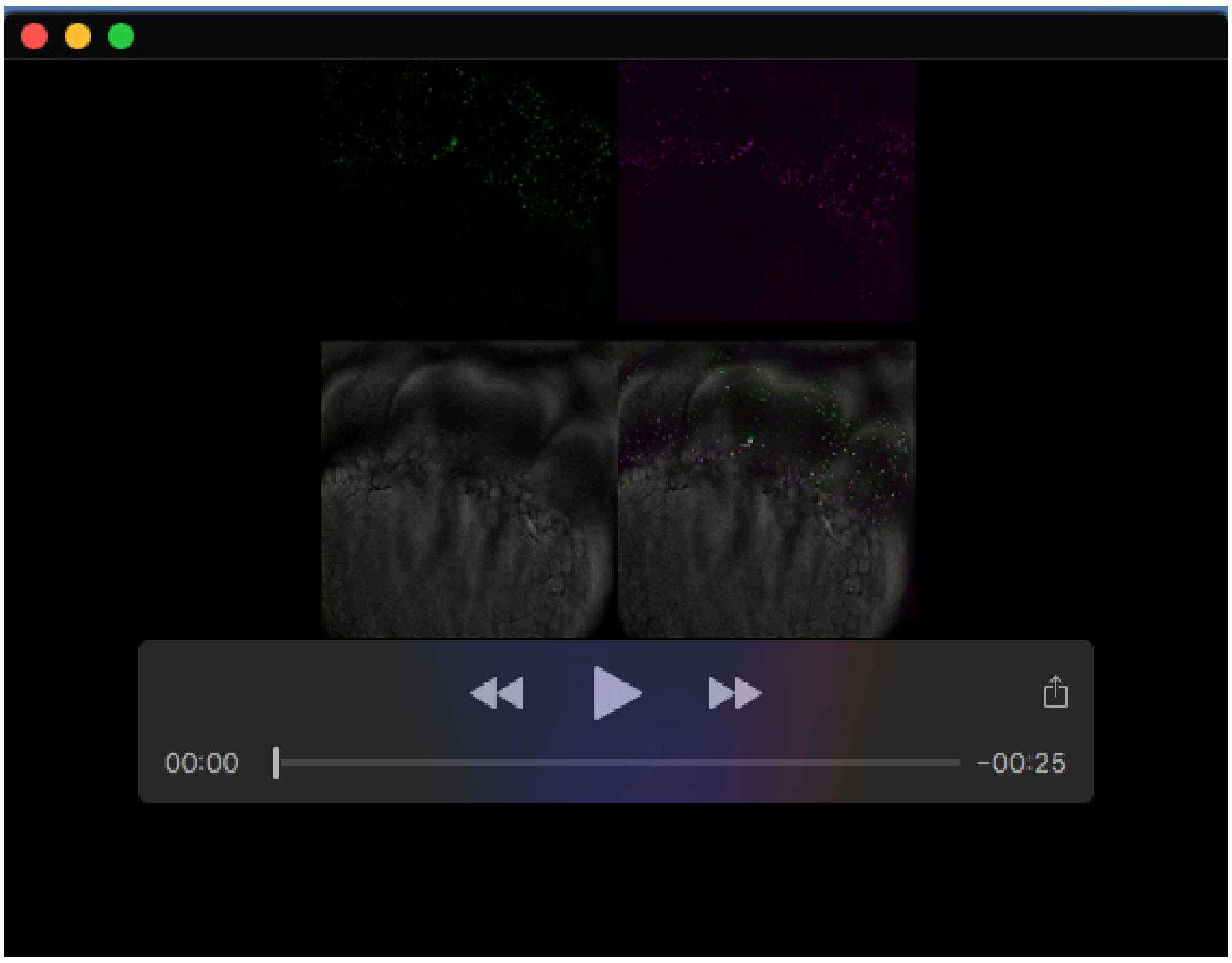

Movie 1. Localization of Tdrd6b-mKate2 during embryogenesis.
Live imaging displaying the localization of Tdrd6b-mKate2 (magenta) throughout the first 3 hours of development. In green Buc-eGFP marking the Gp.

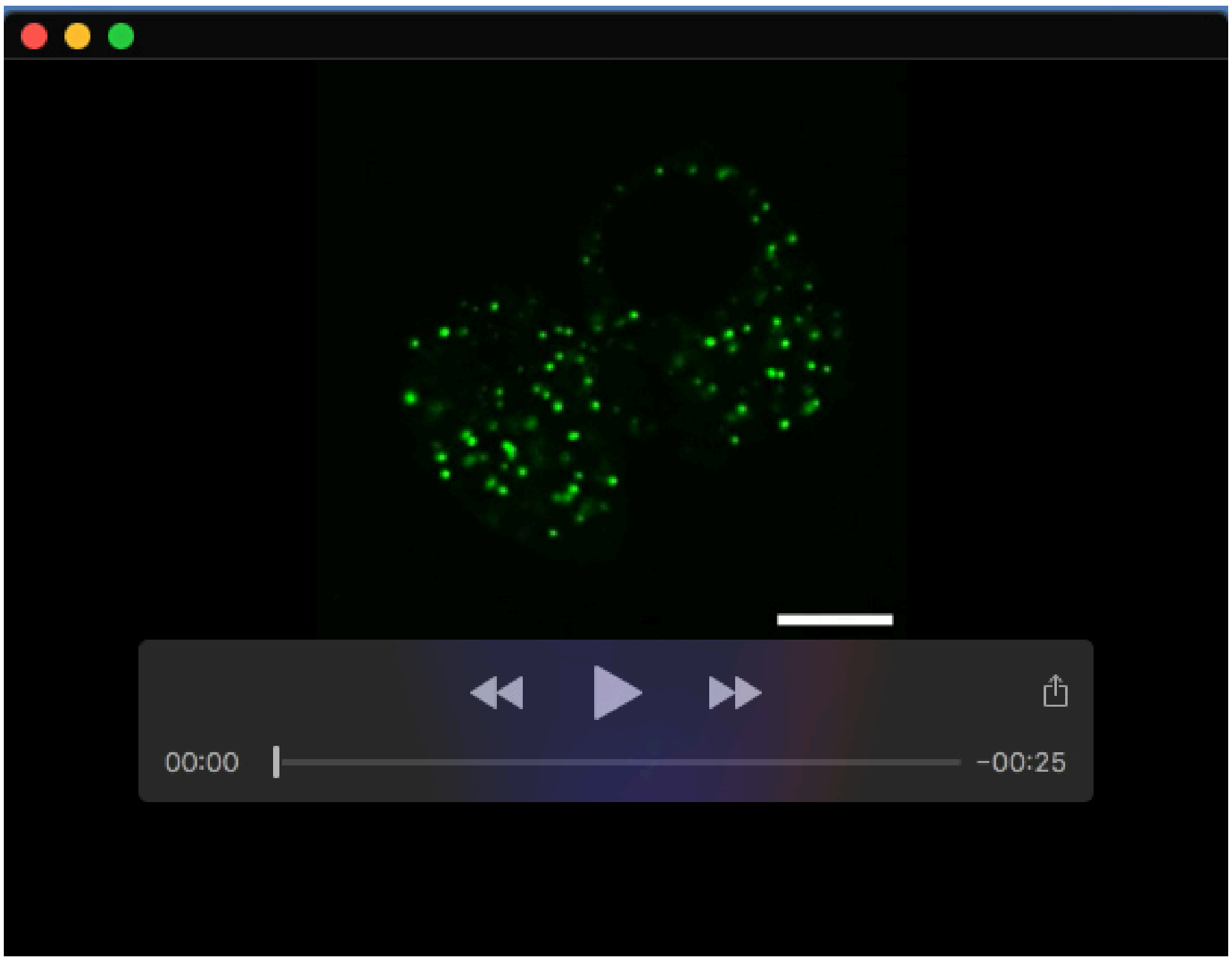

Movie 2. BucGFP behavior in BmN4 cells.
Live imaging of Buc-GFP (green) expressed in BmN4 cells (time-lapse of 3 minutes). Scale bar = 10 µm.

Development • Supplementary information

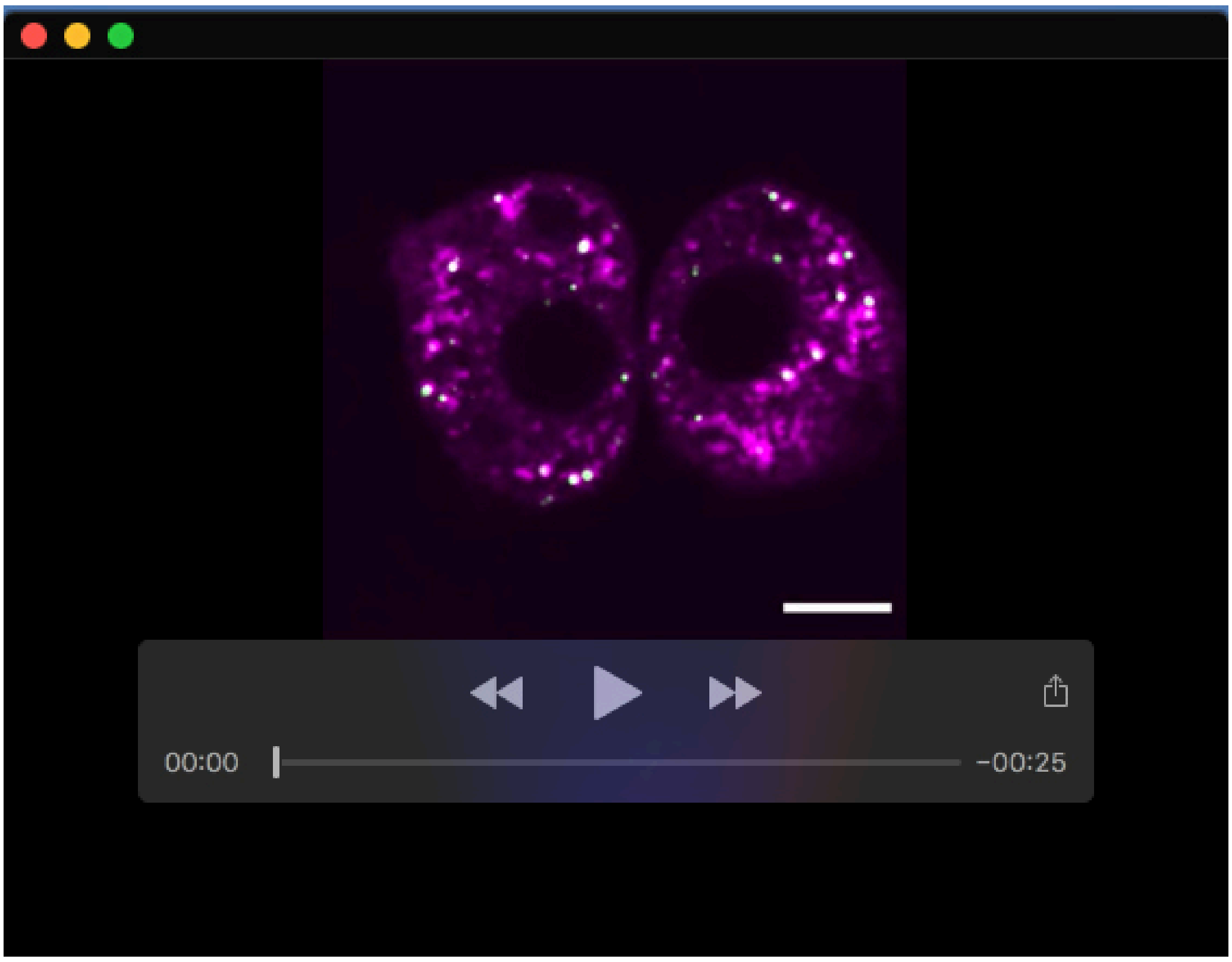

Movie 3. BucGFP behavior in BmN4 cells co-expressing mCherry-PrLD.

Live imaging of Buc-eGFP (green) expressed in BmN4 cells together with mCherry-PrLD (magenta) (time-lapse of 12 hours). Scale bar = 10 µm
